# Antifungal Efficacy and Chemical Profiles of *Salvia*, *Oregano*, and *Laurus* Essential Oils Against Mold Species Associated with Caciocavallo Cheese Spoilage

**DOI:** 10.3390/molecules31193439

**Published:** 2026-09-27

**Authors:** Shazia Akram, Beatrice Brandimarte, Giuseppe Natale Basile, Giuseppe De Mastro, Clemencia Chaves Lopez

**Affiliations:** 1 Department of Bioscience and Technology for Food, Agriculture and Environment, University of Teramo, Via R. Balzarini 1, 64100 Teramo, Italy; bbrandimarte@unite.it; 2 Department of Soil, Food and Plant Sciences, University of Bari Aldo Moro, 70126 Bari, Italy; giuseppe.basile@uniba.it (G.N.B.); giuseppe.demastro@uniba.it (G.D.M.)

**Keywords:** Caciocavallo, essential oils, antifungal activity, spoilage molds, food safety

## Abstract

Mold contamination is a major cause of spoilage in ripened cheeses. In this study, 171 fungal isolates were recovered from semi-hard Caciocavallo cheese sampled across three seasons. The number of recovered isolates showed a descriptive decrease from summer to winter. Thirty-two representatives were selected for ITS and/or β-tubulin sequencing after morphotype screening. Among these sequenced representatives, *Penicillium commune* was the most frequent provisional taxonomic designation (8/32; 25.0%), followed by *Aspergillus flavus* (3/32; 9.4%). *Penicillium echinulatum*, *Fusarium iranicum*, *Paecilomyces variotii*, and Cladosporium-like isolates each accounted for 2/32 (6.3%). ITS-only assignments in taxonomically difficult genera are treated as provisional closest-match designations rather than definitive species identifications. Essential oils (EOs) from three *Salvia rosmarinus* Spenn. chemotypes (SR-CV2-23, SR-CV3-23, and SR-CV6-23), oregano cv. Carva (*Origanum vulgare* L. subsp. *viridulum* (Martrin-Donos) Nyman × *O. vulgare* L. subsp. *hirtum* Ietswaart; Ov-C-23), and *Laurus nobilis* L. (Ln-23) were chemically characterized and evaluated for their antifungal activity against mold species associated with Caciocavallo cheese spoilage. GC-MS showed that the rosemary EOs were rich in camphor, α-pinene, and 1,8-cineole; Ov-C-23 contained 83.95% carvacrol; and Ln-23 contained mainly 1,8-cineole, linalool, and bornyl acetate. All EOs exhibited concentration-dependent antifungal activity, with spore germination generally more sensitive than mycelial growth. At 0.35% (*v*/*v*), SR-CV2-23 showed the broadest observed in-vitro activity, whereas Ov-C-23 produced the highest observed inhibition of *A. alternata* mycelial growth. CFDA/PI confocal microscopy showed the largest relative shift toward reduced metabolic activity and membrane compromise after SR-CV3-23 treatment. In-vitro assays and cheese-slice experiment used different quantitative endpoints and were therefore not directly compared. Ov-C-23 produced the highest observed reduction in visible colonies in the cheese slice model. These EOs may contribute to integrated mold control strategies, but practical application requires further validation under industrial conditions.

## 1. Introduction

Cheese is one of the most widely consumed dairy products and is an important source of high-quality proteins, vitamins, and minerals. Among traditional Italian cheeses, Caciocavallo is a pasta filata cheese of considerable technological and commercial importance [1]. It is generally produced from cow’s milk using natural whey cultures or commercial thermophilic starters, mainly *Streptococcus thermophilus* and *Lactobacillus helveticus*, sometimes combined with mesophilic cultures such as Lactococcus lactis [2]. Caciocavallo undergoes a maturation period of at least two months, while traditional products may ripen for four months to one year. During ripening and storage, filamentous fungi can colonize cheese surfaces, reduce sensory quality and shelf life, and, depending on species and strain, produce mycotoxins. *Aspergillus flavus* is associated primarily with aflatoxins, whereas *Penicillium commune* may produce cyclopiazonic acid; other cheese-associated *Penicillium* species may produce citrinin (CIT) or ochratoxin A (OTA). Maintaining microbiological stability therefore remains important for the producers, regulators, and consumers [3,4,5,6]. Increasing demand for clean-label foods has stimulated interest in EOs as sources of antimicrobial compounds [7,8]. Their activity, however, depends strongly on chemical composition, which varies with genotype, environment, developmental stage, plant tissue, and extraction conditions [9]. EOs can inhibit fungi through membrane disruption, increased cellular permeability, and interference with essential metabolic processes [7,8]. However, cheese proteins and lipids can bind or partition hydrophobic constituents, thereby reducing their freely available concentration [9]. Moreover, intense aroma and flavor may limit the EO dose that remains acceptable from a sensory perspective. These constraints require chemical characterization and matrix-specific validation before EOs can be considered for practical cheese preservation. Although the antifungal activity of rosemary, oregano, and laurel EOs is well documented in laboratory media, evidence obtained with wild molds from Caciocavallo cheese and within this specific lipid protein matrix remains limited. The cheese matrix can alter EO partitioning, diffusion, volatility, and freely available concentration. Accordingly, this study aimed to (i) describe the culturable fungal population recovered from Caciocavallo cheese across three sampling seasons; (ii) characterize EOs from SR-CV2-23, SR-CV3-23, and SR-CV6-23, Ov-C-23, and Ln-23 by GC-MS; and (iii) evaluate their antifungal activity against wild cheese-associated molds in vitro and in an artificially inoculated cheese slice model.

The novelty lies in the combined evaluation of chemically characterized oils against wild Caciocavallo isolates using a cheese slice matrix model, rather than in a claim that EO activity in cheese is unprecedented.

## 2. Results

### 2.1. Seasonal Distribution of Isolated Fungal Genera

A total of 171 culturable fungal isolates were recovered from Caciocavallo cheese samples collected across three seasons. Table 1 presents the number and descriptive frequency (%) of filamentous fungal isolates relative to the total recovered in each season. These data describe fungal diversity and distribution and should not be interpreted as independent sample-level abundance. Consequently, the seasonal pattern is presented as a descriptive trend rather than as a statistically significant difference based on pooled isolate counts. The highest number of isolates was recovered in summer (70), followed by autumn (61) and winter (40), indicating a gradual descriptive decrease from the warmer to the colder seasons. In summer, *Penicillium* spp. was the most frequent genus (28.57%), followed by *Alternaria* spp. (17.14%), *Fusarium* spp. (14.29%), and *Paecilomyces* spp. (12.86%). *Aspergillus* spp. and *Cladosporium* spp. each accounted for 11.43%, *Microascus* spp. accounted for 4.29%. In autumn, *Penicillium* spp. remained the most frequent genus (27.87%), closely followed by *Fusarium* spp. (26.23%). *Paecilomyces* spp. represented 18.03%, *Aspergillus* spp. 16.39%, *Alternaria* spp. 8.20%, and *Microascus* spp. 3.28%; *Cladosporium* spp. was not detected. In winter, *Fusarium* spp. was the most frequent genus (25.00%). *Penicillium* spp. and *Paecilomyces* spp. each represented 22.50%, followed by *Aspergillus* spp. (17.50%) and *Alternaria* spp. (10.00%). *Cladosporium* spp. occurred at a low frequency (2.50%), whereas *Microascus* spp. was not detected.

Overall, *Penicillium* spp. predominated in summer and autumn, whereas *Fusarium* spp. was the most frequent genus in winter (Figure 1). Across all 171 isolates, *Penicillium* spp. represented the largest proportion of the fungal population, followed by *Fusarium* spp., *Paecilomyces* spp., *Aspergillus* spp., *Alternaria* spp., *Cladosporium* spp., and *Microascus* spp. The descriptive seasonal distribution also varied among individual groups: *Cladosporium* spp. was detected in summer and winter but not in autumn, whereas *Microascus*-like isolates were recovered only in summer and autumn.

### 2.2. Molecular Identification and Taxonomic Diversity of Representative Fungal Isolates

The 171 isolates were grouped by macroscopic colony traits and microscopic reproductive structures. Thirty-two representatives were then selected to verify recurring morphotypes by ITS and/or β-tubulin sequencing. The sequenced collection comprised *Penicillium*, *Aspergillus*, *Fusarium*, *Alternaria*, *Cladosporium*-like, *Paecilomyces*, and *Microascus*-like taxa (Table 2; Figure 2).

*Penicillium* was the most diverse genus. Following molecular identification, representative sequences were analyzed using the NCBI BLASTn web tool against the GenBank database. The 32 sequenced representatives included eight *P. commune* assignments, three *A. flavus* assignments, two *P. echinulatum* assignments, two *F. iranicum* assignments, two *P. variotii* assignments, and two *Cladosporium*-like isolates. Single representatives were assigned to *P. polonicum*, *P. brevicompactum*, *P. solitum*, *P. crustosum*, *P. camemberti*, *P. digitatum*, *P. discolor*, *A. montevidensis*, *F. tricinctum*, *F. avenaceum*, *A. alternata*, and *A. tenuissima*, together with one *Microascus*-like isolate (Figure 2). Because several taxonomically difficult genera were represented only by ITS sequences, these species-level names are presented as provisional closest-match designations and are not intended to imply definitive multi-locus species identification.

Species level identification was not assigned to the *Cladosporium*-like and *Microascus*-like isolates because sequence similarity was insufficient for confident discrimination, particularly for the *Microascus*-like isolate (91.25%). Mo re generally, ITS-only assignments in *Penicillium*, *Aspergillus*, *Fusarium*, and *Alternaria* are interpreted conservatively as provisional closest-match designations. Additional loci and phylogenetic analyses using authenticated reference strains would be required for definitive taxonomic resolution. Table 2 reports the selected GenBank reference match used for each BLAST-based assignment and the total number of isolates associated with the corresponding morphotype group.

### 2.3. Comparison of Mycelial Growth on CAM and PDA

Following the optimization of the cheese agar medium (CAM), the selected formulation was compared with standard potato dextrose agar (PDA) at 14 ± 2 °C. Two-way ANOVA results revealed significant “isolate × medium” interactions for both *Penicillium* isolates [F(9,20) = 2.82, *p* = 0.0257] and other fungal isolates [F(9,20) = 8.83, *p* < 0.0001] (Figure 3). Simple-effect comparisons with Sidak correction showed no significant difference between CAM and PDA for any of the *Penicillium* isolates. Among the other fungi, *F. iranicum*, *F. tricinctum*, and *F. avenaceum* exhibited significantly better growth on PDA compared to CAM (adjusted *p* < 0.0001, *p* = 0.0348, and *p* = 0.0348, respectively), whereas the other isolates did not show a significant difference depending on the medium. Overall, CAM supported the growth of various cheese-associated molds at 14 ± 2 °C.

### 2.4. Essential Oil Chemical Composition

Essential oil yield varied among the investigated plant species and cultivars (Table 3). The highest yield was recorded for the rosemary chemotype SR-CV2-23 (3.33% *w*/*w* on a dry-weight basis), followed by SR-CV3-23 (2.71%), oregano Ov-C-23 (2.65%), and SR-CV6-23 (2.25%). Bay laurel Ln-23 produced the lowest yield (1.08%). Among the rosemary chemotypes, yields ranged from 2.25% to 3.33%, indicating substantial variation despite cultivation under the same environmental and agronomic conditions.

The major chemical constituents of the essential oils from the *Salvia rosmarinus* chemotypes (SR-CV2-23, SR-CV3-23, and SR-CV6-23), oregano cv. Carva (Ov-C-23), and *Laurus nobilis* (Ln-23), as determined by GC-MS, are presented in Table 4. In the three *S. rosmarinus* essential oils (SR-CV2-23, SR-CV3-23, and SR-CV6-23), 32, 15, and 23 compounds were identified, respectively. Camphor, α-pinene, and 1,8-cineole were the principal constituents, although their relative proportions differed among samples. SR-CV2-23 contained predominantly camphor (37.83%), α-pinene (20.82%), and 1,8-cineole (13.29%). In SR-CV3-23, camphor was also the major compound (47.84%), followed by α-pinene (17.81%) and 1,8-cineole (9.26%). SR-CV6-23 contained camphor (23.81%), α-pinene (17.42%), and 1,8-cineole (11.35%). Thus, the three rosemary *essential* oils had related but distinct compositional profiles. The oregano essential oil (Ov-C-23) contained 26 identified compounds and was dominated by carvacrol (83.95%), with lower proportions of p-cymene (3.28%), γ-terpinene (3.25%), and trans-caryophyllene (2.61%). In *L. nobilis* essential oil (Ln-23), 35 compounds were detected; the most abundant were 1,8-cineole (16.87%), linalool (14.71%), bornyl acetate (10.73%), sabinene (7.23%), and eugenol (7.07%). The EO profiles therefore varied both among plant species and among the three *S. rosmarinus* chemotypes, possibly reflecting differences in plant origin, harvest period, environmental conditions, and the plant material used for extraction. Complete constituent lists and relative percentages are provided in Supplementary Tables S1–S3.

### 2.5. Inhibitory Effects of Essential Oils on Mycelial Growth and Spore Germination of Wild Fungal Strains

All tested EOs exhibited concentration-dependent antifungal activity against the eight fungal taxa. Inhibition was generally highest at 0.35% (*v*/*v*) and decreased with concentration. Supplementary Tables S4 and S5 provide complete concentration-response data as mean ± SE from three independent experiments, whereas Table 5 and Table 6 summarize the highest observed numerical inhibition for each taxon and response variable. For the *S. rosmarinus* cultivars at 0.35%, SR-CV2-23 showed high observed mycelial inhibition against *P. camemberti* (53.0 ± 2.0%), *P. commune* (56.0 ± 2.0%), *A. alternata* (51.0 ± 2.0%), *A. tenuissima* (48.3 ± 3.7%), and *F. tricinctum* (42.1 ± 2.0%). SR-CV6-23 showed 34.0 ± 5.9% inhibition against *F. avenaceum* and 44.7 ± 2.7% against *A. montevidensis*, whereas SR-CV3-23 reached 48.3 ± 3.7% against *A. flavus*. Spore-germination inhibition was generally greater than mycelial-growth inhibition; observed maxima included 73.0 ± 3.0% for *A. montevidensis* and 73.0 ± 6.0% for *A. flavus* with SR-CV2-23, 73.0 ± 3.0% for *A. montevidensis* with SR-CV6-23, 71.0 ± 5.0% for *A. tenuissima* with SR-CV3-23, and 69.5 ± 3.5% for *A. alternata* with SR-CV6-23. These numerical maxima are not interpreted as statistically different unless supported by the ANOVA framework described in Section 4.11.

Ov-C-23 produced the highest observed mycelial-growth inhibition against *A. alternata* (58.5 ± 4.4%) at 0.35% (Table 6). Ln-23 showed higher observed effects on spore germination than on mycelial growth, with 71.0 ± 6.0% inhibition for *P. camemberti*, 71.0 ± 3.0% for *A. alternata*, 64.5 ± 3.5% for *F. tricinctum*, and 64.0 ± 2.0% for *A. tenuissima* at 0.35%. Concentration-dependent responses at lower doses are provided in Supplementary Table S5. The terms “highest” and “greater” in this section refer to observed numerical values unless an inferential comparison is explicitly stated.

The heat map in Figure 4 illustrates species-specific susceptibility patterns among the tested fungi and EOs. The greatest overall antifungal activity, considering both mycelial-growth and spore-germination inhibition, was observed against *A. montevidensis*, *A. flavus*, *A. alternata*, and *P. commune*, with maximum inhibition values of 66.0-73.0%. SR-CV2-23, SR-CV3-23, SR-CV6-23, and Ln-23 produced the highest overall inhibition, whereas *F. tricinctum* and *F. avenaceum* were generally less susceptible. These results show that antifungal efficacy depended on both fungal species and EO source.

### 2.6. Antifungal Effect of Essential Oils on Spore Viability in the Representative Wild Strain P. commune

*Penicillium commune* was selected for the viability assay because it was among the most abundant fungi isolated from Caciocavallo cheese and is an important spoilage species with toxigenic potential. Confocal microscopy combined with CFDA/PI staining revealed clear differences in *P. commune* spore viability after treatment with 0.35% (*v*/*v*) EOs (Figure 5). Untreated spores showed strong green CFDA fluorescence and minimal red PI fluorescence, indicating high metabolic activity and intact cell membranes. In contrast, EO-treated spores showed lower CFDA fluorescence and higher PI fluorescence, indicating membrane damage and loss of metabolic activity.

EO-treated spores showed a qualitative shift toward reduced green fluorescence and increased red fluorescence, indicating reduced metabolic activity and increased membrane permeability. Among the tested treatments, SR-CV3-23 visually produced a pronounced shift toward PI-associated red fluorescence, while Ov-C-23 also produced evident membrane-compromise signals. Ln-23 showed a comparatively less marked visual change. These observations are interpreted qualitatively and provide supportive evidence of EO induced alterations in spore metabolic activity and membrane integrity rather than quantitative estimates of viability.

### 2.7. Essential Oils Inhibit Fungal Growth and Sporulation on Artificially Contaminated Cheese Surfaces

The antifungal effects of the EOs were evaluated on cheese slices artificially inoculated with *P. commune* (Figure 6A). The inoculated slices were incubated at 14 ± 2 °C for two weeks. After 14 days at 14 ± 2 °C, the emulsifier treated inoculated control showed extensive visible colony development. EO-treated samples showed lower visible colony numbers in a concentration- and oil-dependent pattern. Because the quantitative endpoint was the number of visible colonies, no quantitative claim regarding sporulation is made.

As shown in Figure 6B, the highest observed reduction in visible colonies was obtained with Ov-C-23, reaching 97.2% at 0.35% and 93.4% at 0.25%. SR-CV2-23 produced reductions of 92.3% and 84.5% at the same concentrations. SR-CV3-23 and SR-CV6-23 showed intermediate numerical reductions, whereas Ln-23 showed lower reductions. These values describe reduction in visible colony counts during experimental study relative to the emulsifier treated control and should not be interpreted as fungal eradication or loss of viability.

### 2.8. Preliminary Non-Ingestive Sensory Evaluation

The preliminary non-ingestion olfactory assessment of treated cheese slices at various concentrations (0.35%, 0.25%, 0.15%, 0.075%, and 0.037% *v*/*v*) showed varied aroma perception according to the type and concentration of the essential oil as shown in Figure 7. Overall, samples at lower concentrations received higher liking scores. Among the rosemary chemotypes, SR-CV3-23 and SR-CV6-23 demonstrated relatively favorable aroma scores at lower concentrations, whereas SR-CV2-23 achieved its highest score at a concentration of 0.25% (*v*/*v*). Aroma scores for Ov-C-23 and Ln-23 also fluctuated with concentration, indicating that olfactory perception is dependent on concentration. These observations are considered preliminary indicators of relative aroma acceptability rather than conclusive evidence of consumer preference, overall product quality, or market acceptance.

## 3. Discussion

The present study confirms that Caciocavallo cheese harbors a diverse community of filamentous fungi. The predominance of *Penicillium*, *Aspergillus*, *Fusarium*, *Alternaria*, and *Paecilomyces* is consistent with previous reports identifying these genera as major spoilage molds in dairy products [5,6]. Nevertheless, the observed distribution also reflects the specific ecological conditions of this pasta filata cheese. *Penicillium* was the most abundant genus, in agreement with studies of Gouda and Cheddar cheeses [10]. *Penicillium* commune is a common spoilage agent of hard and semi-hard cheeses and may produce cyclopiazonic acid [11,12]. The frequent recovery of *Aspergillus*, particularly *A. flavus*, together with *Fusarium*, *Alternaria*, Paecilomyces, and Cladosporium, suggests that the Caciocavallo mycobiota reflects both ripening-room conditions and broader environmental contamination rather than exclusively dairy adapted taxa.

The total number of recovered isolates decreased descriptively from summer to winter (70 to 40 isolates). Because pooled isolate counts were used, this pattern should not be interpreted as a statistically significant seasonal effect. It may nevertheless reflect differences in temperature, humidity, environmental fungal load, and air circulation in traditional cheese-production facilities. *Penicillium* predominated in summer and autumn, whereas *Fusarium* was most frequent in winter. *Cladosporium* and *Alternaria* also decreased during the colder period, consistent with reported seasonal patterns of airborne fungal spores [13,14]. Understanding seasonal influences and resident environmental populations is important for optimizing production protocols and limiting spoilage [15]. The comparison between PDA and the optimized CAM further supported the value of a cheese-based screening medium. Several isolates, particularly *Penicillium* species, grew well on Cheese agar medium at 14 ± 2 °C, suggesting that this medium reproduces some of the nutritional and physicochemical constraints of cheese. Because conventional media may not accurately predict mold behavior in complex food matrices [16], CAM may improve the relevance of EO screening for Caciocavallo cheese. These observations provide a descriptive account of seasonal variation in the culturable surface mycobiota recovered from Caciocavallo cheese.

EO efficacy should be interpreted in relation to chemical composition rather than plant species alone [8]. The three *S. rosmarinus* essential oils differed in the relative proportions of camphor, α-pinene, and 1,8-cineole, which may explain their distinct antifungal profiles [17]. Ov-C-23 was strongly dominated by carvacrol, confirming its carvacrol-rich profile [18].

Ln-23 showed a 1,8-cineole/linalool-rich profile, with bornyl acetate as the third main identified components. The identified compounds accounted for 84.25% of the chromatographic area; therefore, interpretation of Ln-23 biological activity should also recognize the 15.75% unidentified fraction. The variable efficacy of the three rosemary cultivars highlights the need for compositional standardization before industrial application [19,20].

All tested EOs exhibited concentration--dependent antifungal activity, and spore germination was generally more sensitive than mycelial growth, consistent with previous reports of EO-mediated inhibition of fungal growth and development [21,22,23]. In vitro assay and cheese-slice experiment used different quantitative endpoints and were therefore not directly compared. Because Ov-C-23 was rich in carvacrol, this composition may have contributed to the observed activity; however, this interpretation remains hypothetical because pure carvacrol and reconstituted mixtures were not tested [24,25,26].

In contrast, the three *S. rosmarinus* EOs showed more heterogeneous and isolate-specific antifungal activity, consistent with variation in camphor, α-pinene, and 1,8-cineole content [27,28]. *Laurus nobilis* EO showed comparatively weak inhibition of mycelial growth, despite previously reported activity in liquid or synthetic media [29,30].

The cheese-slice experiments were performed under controlled laboratory conditions and do not fully reproduce industrial ripening environments. Although *P. commune* visible colony development was markedly reduced, complete inhibition or loss of viability was not demonstrated. The experiment quantified visible colonies rather than sporulation or toxin production; therefore, the data should not be used to infer reduced sporulation or mycotoxin accumulation [31,32].

In the in vitro assays, mycelial-growth and spore-germination inhibition generally fell within an approximate 30–75% range across many oil taxon combinations, whereas the cheese-slice experiment used a different endpoint and reached up to 97.2% reduction in visible colony counts. These endpoints should not be directly equated. The cheese-slice values indicate fewer visible colonies relative to the control, not near-complete fungal eradication or loss of viability. Direct use of EOs in a complex food matrix must also satisfy sensory, technological, regulatory, and economic constraints.

Growth inhibition and fungal secondary metabolism are not necessarily proportional. In toxigenic species such as *A. flavus* or *P. commune*, reduced biomass or visible colony formation cannot be equated with reduced mycotoxin biosynthesis [10,23,33,34]. Because mycotoxins were not measured in this study, any antimycotoxigenic interpretation remains outside the present evidence and requires direct analytical quantification.

A further practical concern is the possible effect of EOs on beneficial lactic acid bacteria (LAB). Although selected EOs can be compatible with some starter cultures at low concentrations, sensitivity varies among strains and formulations [35,36,37,38]. Future studies should therefore assess fungal inhibition together with starter-culture viability, acidification kinetics, ripening performance, and sensory quality. Regulatory status also differs by jurisdiction: in the United States, certain essential oils, oleoresins, and natural extractives are listed under 21 CFR 182.20 [39], and natural flavoring substances are addressed under 21 CFR 172.510 [40]; in the European Union, flavorings are regulated under Regulation (EC) No. 1334/2008 [41]. These provisions do not by themselves establish authorization of an EO as a cheese preservative, so intended use, composition, dose, and applicable food-category requirements must be evaluated before commercialization.

The CFDA/PI viability assay provided qualitative complementary evidence of EO associated changes in metabolic activity and membrane integrity. EO-treated spores showed a visual shift toward reduced CFDA associated green fluorescence and increased PI-associated red fluorescence. These observations are interpreted qualitatively and not as quantitative estimates of viability.

Overall, the results show that selected plant EOs reduced mycelial growth, spore germination, relative viability signals, and visible colony development of cheese-associated molds. Efficacy depended on oil composition, concentration, fungal taxon, and the cheese matrix. The absence of MIC/MFC or EC50 determinations and of a commercial antifungal comparator, such as natamycin, limits direct benchmarking. Reduced visible growth should not be interpreted as reduced mycotoxin production because toxins were not quantified. These findings support further evaluation of EOs as components of integrated preservation systems rather than as replacements for hygiene, packaging, temperature control, or established preservatives.

## 4. Materials and Methods

### 4.1. Isolation of Molds from Caciocavallo Cheese Samples

Caciocavallo cheese samples were collected from the same Caciocavallo cheese provided by the Capurso Azienda Casearia S.r.l. (Gioia del Colle, Italy), during three different seasons: summer (July), autumn (October), and winter (January). Three biologically independent cheese samples were analyzed from the same production source at each sampling period. The same sampling procedure was used throughout the study, and the cheese type and handling conditions were kept consistent; therefore, season represented the main difference among the three sampling periods. The sampling design was intended to describe seasonal variation in the culturable fungal population from this production source and not to compare different producers or production environments. Ten grams of each sample was homogenized in 90 mL of sterile physiological solution, serially diluted, and plated on potato dextrose medium (PDA), yeast dextrose medium (YPD) obtained from Oxoid Limited, Thermo Fisher Scientific (Basingstoke, Hampshire, UK), Rose Bengal (RB) Sigma-Aldrich/Merck (Darmstadt, Germany), and cheese agar medium supplemented with chloramphenicol obtained from Sigma-Aldrich/Merck (Darmstadt, Germany). Plates were incubated at 14 ± 2 °C for 7 days. Representative fungal colonies were selected from the recovered population, purified on PDA, and preserved at 4 °C for further morphological and molecular identification.

### 4.2. Identification of Fungi

All 171 isolates were screened using macroscopic colony characteristics (color, texture, margin, reverse pigmentation, and sporulation) and microscopic reproductive structures, following Samson et al. [42], and assigned to recurring morphotype groups. Thirty-two representatives spanning these groups were selected for molecular analysis. Table 2 reports each sequenced representative and the number of morphologically similar isolates associated with it. Species-level percentages shown in Figure 2 are based only on the 32 sequenced representatives; genus-level seasonal counts in Table 1 are based on all 171 recovered isolates.

### 4.3. Molecular Identification of Molds Based on ITS and β-Tubulin Gene Analysis

Following microscopic characterization, isolates were identified using the internal transcribed spacer (ITS) region and/or β-tubulin gene markers [43,44]. Pure cultures were grown in yeast dextrose broth for 5 days at 25 °C in a shaker. Mycelium was transferred to sterile eppendorf tubes, centrifuged at 5000 rpm for 15 min, and washed three times with sterile physiological solution. Approximately 100 mg wet biomass was used for DNA extraction by using a Zymo DNA extraction kit (Zymo Research, Irvine, CA, USA) according to the manufacturer’s protocol with minor modifications. DNA quality and concentration were assessed using a Bio-Rad DNA quantification instrument (Bio-Rad Laboratories, Hercules, CA, USA). Primer pairs and PCR conditions are summarized in Table 7.

PCR products were separated on 2% agarose gels and visualized using Gel Doc system (Alpha Innotech Corp., San Leandro, CA, USA). Purified amplicons were sequenced by BMR Genomics S.r.l. (Padova, Italy). Consensus sequences were compared with the GenBank database using the NCBI BLASTn web tool. Because the accession numbers listed in Table 2 correspond to the closest GenBank matches used for taxonomic assignment, the table heading has been revised accordingly; they should not be interpreted as accession numbers assigned to the sequences generated in this study. The consensus sequences should be deposited in a public repository before final acceptance; until accession numbers are assigned, the closest-match accessions are reported transparently.

### 4.4. Preparation of Modified Cheese Agar Medium (CAM) and Comparison of Mycelial Growth on CAM and PDA

Caciocavallo agar medium (CAM) was prepared according to Akram et al. [16] with minor modifications to mimic the cheese matrix and support the growth of cheese-associated molds. Representative isolates from dominant and recurrent taxa were inoculated onto CAM and PDA using the same standardized inoculum plug size, plate, medium volume, incubation temperature (14 ± 2 °C), and 12 days duration. Mycelial growth was evaluated by comparing colony diameter on both media.

### 4.5. Collection of Plant Material, Extraction, Chemical Characterization

Oregano cv. Carva (*Origanum vulgare* L. subsp. *viridulum* (Martrin-Donos) Nyman × *O. vulgare* L. subsp. *hirtum* Ietswaart; Ov-C-23) was cultivated at the Experimental Educational Centre “E. Pantanelli”, University of Bari Aldo Moro (40°10′18.0″ N, 16°38′57.2″ E; Policoro, Matera, Southern Italy). Seeds were supplied by MediSeeds SA (Conthey, Switzerland), and seedlings were transplanted in double rows on biodegradable mulch under drip irrigation. Plants were harvested at the balsamic stage, corresponding to early-full flowering, in June 2024. Bay laurel (*Laurus nobilis* L.; Ln-23) leaves were collected between September and October 2024 from mature trees growing in a wooded area within the same experimental centre. Rosemary (*Salvia rosmarinus* Spenn.) chemotypes SR-CV2-23, SR-CV3-23 and SR-CV6-23 were sampled from the rosemary germplasm collection maintained at the Experimental Educational Centre “E. Pantanelli” and cultivated under identical agronomic and environmental conditions. The aerial parts were harvested at full flowering.

All plant materials, including oregano cv. Carva (Ov-C-23), *S. rosmarinus* chemotypes SR-CV2-23, SR-CV3-23 and SR-CV6-23, and bay laurel (Ln-23), are maintained as living accessions at the Experimental Educational Centre “E. Pantanelli”. The reference plant material is preserved in vivo as part of the Field Crops Laboratory Living Collection (DiSSPA, University of Bari Aldo Moro, Bari, Italy) for research, breeding and germplasm conservation purposes. Botanical identification of all taxa was performed using diagnostic morphological characters according to *Flora d’Italia* and Euro + Med PlantBase. Accordingly, the living accessions maintained at the Experimental Educational Centre constitute the reference material for this study.

Oregano and rosemary aerial parts were air-dried under shaded and ventilated conditions at temperatures not exceeding 40 °C until constant weight, whereas laurel leaves were dried in a forced-air ventilated oven at 45 °C. The dried material was stored under dry conditions and protected from light until extraction. Essential oils were extracted by steam distillation using an InHerba Inox Plus apparatus (Albrigi Luigi S.r.l., Stallavena, Verona, Italy) installed at the Herbaceous Crops Laboratory of the Department of Soil, Plant and Food Sciences (DiSSPA), University of Bari Aldo Moro, following the procedure described in the European Pharmacopoeia 11th ed.; Council of Europe, Strasbourg, France, 2024. Approximately 100 g of dried plant material was subjected to distillation for 3.5 h, and the process was terminated when no further increase in essential oil volume was observed. Essential oil yield was expressed as percentage (*w*/*w*) on a dry-weight basis. The oil phase was separated from the aqueous distillate, dried over anhydrous sodium sulphate, transferred into amber glass vials and stored at 4 °C until analysis.

The chemical composition of the EOs was determined by GC-MS using an Agilent 7890 gas chromatograph coupled to an Agilent 5975C mass-selective detector and an HP-5MS capillary column (30 m × 0.25 mm i.d.; 0.25 μm film; Agilent Technologies, Santa Clara, CA, USA). The oven was held at 60 °C for 2 min, increased to 110 °C at 2 °C min^−1^ and then to 220 °C at 10 °C min^−1^, with a 10-min final hold. Helium was used at 1.1 mL min^−1^. Samples were diluted 1:100 (*v*/*v*) in n-hexane; 1 μL was injected at a 1:50 split ratio. Spectra were acquired by EI at 70 eV over *m*/*z* 40-400. Linear retention indices were calculated against n-alkanes C8-C32 analyzed under the same conditions and compared with literature data [45]. Mass spectra were matched against the NIST 17 library [46]. Major constituents were confirmed by co-injection with authentic standards when available. Relative abundances were calculated by peak-area normalization without response-factor correction. Complete data, including retention time, experimental and literature indices, identification method, and total identified percentage, are provided in Supplementary Tables S1–S3.

### 4.6. Preparation of Essential Oil Emulsions

EO emulsions were prepared in sterile distilled water containing 0.1% (*v*/*v*) Tween 80 as the sole emulsifying agent. The required volume of each EO was added, and sterile distilled water was then added to the final volume to obtain EO concentrations of 0.35%, 0.25%, 0.15%, 0.075%, and 0.037% (*v*/*v*). The mixtures were vortexed for 5 min and sonicated until homogeneous. The corresponding negative control contained 0.1% (*v*/*v*) Tween 80 but no EO.

### 4.7. Antifungal Activity of Essential Oils Against Selected Cheese Isolated Mold Species

The selected test fungi were *P. camemberti*, *P. commune*, *A. alternata*, *A. tenuissima*, *F. avenaceum*, *F. tricinctum*, *A. montevidensis*, and *A. flavus*. EO emulsions were incorporated directly into CAM at 0.35%, 0.25%, 0.15%, 0.075%, and 0.037% (*v*/*v*), following a contact-assay approach adapted from Grande-Tovar et al. [47]. Mycelial plugs from actively growing colonies were placed centrally on treated CAM. Controls were included: untreated CAM control without EO, while EO-free plates served as controls. Plates were incubated at 14 ± 2 °C until control colonies approached the plate edge. No commercial antifungal positive control was included; this is acknowledged as a limitation.

Mycelial-growth inhibition was calculated as follows:

PI (%) = [(Ac − At)/Ac] × 100, (1)

where Ac is the mean colony diameter in the emulsifier control and At is the mean colony diameter in the EO-treated plate.

For the spore-germination inhibition (SGI) assay, spores from 7-day-old cultures were suspended in sterile distilled water containing 0.1% Tween 80 and adjusted to approximately 1 × 10^6^ spores mL^−1^. Aliquots were exposed to the corresponding EO concentrations and incubated for 24 hpt at 14 ± 2 °C. At least 100 spores were counted across randomly selected microscopic fields in each independent replicate. A spore was considered germinated when its germ tube length equaled or exceeded the smallest spore diameter. Three independent experiments were performed following the general microscopic approach described by Droby et al. [48].

Spore-germination inhibition was calculated as

SGI (%) = [(Gc − Gt)/Gc] × 100, (2)

where Gc and Gt are the percentages of germinated spores in the control and EO-treated sample, respectively.

### 4.8. Effect of EO Treatment on Fungal Cell Viability

Fungal cell viability was assessed by dual staining with 5(6)-carboxyfluorescein diacetate (CFDA) and propidium iodide (PI) (Sigma-Aldrich/Merck, Darmstadt, Germany), followed by observation using a Nikon A1R confocal imaging system (Nikon Corp., Tokyo, Japan). CFDA positive cells were considered viable and metabolically active, whereas PI positive cells were considered membrane damaged or non-viable. To conduct the assay, fungal spores were first harvested from 7-day old mature cultures using sterile phosphate-buffered saline (PBS; pH 7.4). The resulting suspension was filtered to remove any hyphal debris and washed twice to eliminate residual media particles. The cleaned spores were then treated with the selected essential oil (EO) and incubated 16 h post treatment (hpt) at 14 ± 2 °C, while untreated spore suspensions were maintained under the same conditions to serve as controls.

Then, 30 µL of CFDA and PI working solutions were added to each sample, followed by incubation in the dark at 30 °C for 15–20 min. After staining, excess dye was removed by washing twice with PBS, and samples were immediately observed by confocal microscopy. Excitation/emission wavelengths of 495/520 nm and 535/620 nm were used for CFDA and PI, respectively. CFDA-associated green fluorescence was interpreted as an indicator of metabolic activity, whereas PI-associated red fluorescence indicated loss of membrane integrity. The staining assay was used as a qualitative comparative assessment and was not interpreted as an absolute measurement of viable or non-viable spores.

### 4.9. Application of EOs to Artificially Inoculated Cheese Slices

Caciocavallo hard cheese slices were used as the experimental matrix because the whole cheese has a hard rind that is not typically consumed and may limit uniform fungal inoculation and treatment exposure. In commercial practice, this cheese is also marketed as cut portions or slices, in which the inner surface becomes exposed and more susceptible to fungal contamination during handling and storage. Therefore, the use of slices allowed standardized inoculation, uniform application of essential oils, and reproducible evaluation of antifungal activity under conditions relevant to commercial settings [9,31].

Cheese slices approximately 2.5×2.5 cm in size, 1 cm thick and weighing 5 g were prepared aseptically and placed individually in sterile Petri dishes. Each slice was surface inoculated with 100 μL of a *P. commune* suspension containing 1 × 10^5^ spores and allowed to dry under sterile conditions. Subsequently, 100 μL of EO emulsion was distributed over the inoculated surface at 0.35%, 0.25%, 0.15%, 0.075%, or 0.037% (*v*/*v*). Emulsifier-treated inoculated slices without EO served as controls. Petri dishes were sealed with parafilm and incubated at 14 ± 2 °C for 14 days and sealed dishes maintained a humid microenvironment. Visible colonies were counted at the end of the incubation period and documented photographically, and colony reduction was subsequently calculated [16].

Colony reduction (%) = [(Cc − Ct)/Cc] × 100, (3)

where Cc is the number of visible colonies counted on the untreated inoculated control slice and Ct is the number of visible colonies counted on the EO-treated slice.

### 4.10. Sensory Analysis

A preliminary non ingestive olfactory screening was performed on non-inoculated Caciocavallo cheese slices treated with SR-CV2-23, SR-CV3-23, SR-CV6-23, Ov-C-23, or Ln-23 at 0.35, 0.25, 0.15, 0.075, and 0.037% (*v*/*v*); untreated slices served as controls. After 24 h of storage at 14 ± 1 °C, untrained adult assessors evaluated randomly coded samples presented in randomized order. The samples were assessed by smell only; no samples were tasted or consumed. Aroma acceptability was scored using a 9-point hedonic scale ranging from 1 (dislike extremely) to 9 (like extremely), following the general approach described by Lim [49].

Water was provided between samples to minimize residual sensory interference. This assessment was intended only as a preliminary screening to support concentration selection for further study. Therefore, the obtained scores were interpreted as descriptive indicators of relative aroma tolerance under the test conditions and not as measures of consumer preference, overall product quality, or market-level acceptability.

### 4.11. Statistical Analysis

Three independent experimental replicates were performed, and results are reported as mean ± SE. Seasonal isolate counts, species composition, EO yield, and GC–MS profiles were analyzed descriptively. CAM/PDA growth data were analyzed by two-way ANOVA including isolate, medium, and the isolate × medium interaction; significant interactions were followed by Sidak-adjusted simple-effect comparisons of CAM versus PDA within each isolate. EO inhibition data were analyzed by two-way ANOVA including EO, concentration, and the EO × concentration interaction, with significant interactions examined by adjusted simple-effect comparisons and reported numerically rather than by compact-letter groupings. CFDA/PI fluorescence images were evaluated qualitatively and were not subjected to inferential statistical analysis. Statistical significance was set at *p* < 0.05. Analyses were performed using Statistix 8.1 and GraphPad Prism 10.3.1.

## 5. Conclusions

This study describes a seasonal pattern in the culturable fungal population recovered from Caciocavallo cheese, with fewer isolates obtained in winter than in summer and a shift from *Penicillium* predominance in summer and autumn to *Fusarium* predominance in winter. Because the analysis was based on pooled recovered isolates, this pattern is descriptive. The tested essential oils (EOs) caused concentration-dependent reductions in mycelial growth, spore germination, relative viability signals, and visible sporulation. Among them, SR-CV2-23 showed broad in vitro activity, while Ov-C-23 produced the highest observed reduction in visible colonies in the cheese slice model. Although the complete eradication was not achieved, and activity depended on oil composition, dose, fungal species, and cheese matrix. The absence of MIC/MFC or EC50 determination, commercial positive control, industrial ripening validation, direct mycotoxin measurements, and a formal consumer sensory study limit practical interpretation. EO-based strategies should therefore be evaluated as components of hurdle technology rather than as standalone preservatives together with appropriately designed sensory studies to assess their practical acceptability.

Future work should quantify mycotoxins directly, benchmark EO treatments against approved antifungal agents such as natamycin, assess effects on starter and ripening microbiota, formulation stability and migration, and validate efficacy and sensory acceptance in formally designed studies conducted under the appropriate ethical and safety framework.

## Figures and Tables

**Figure 1:**
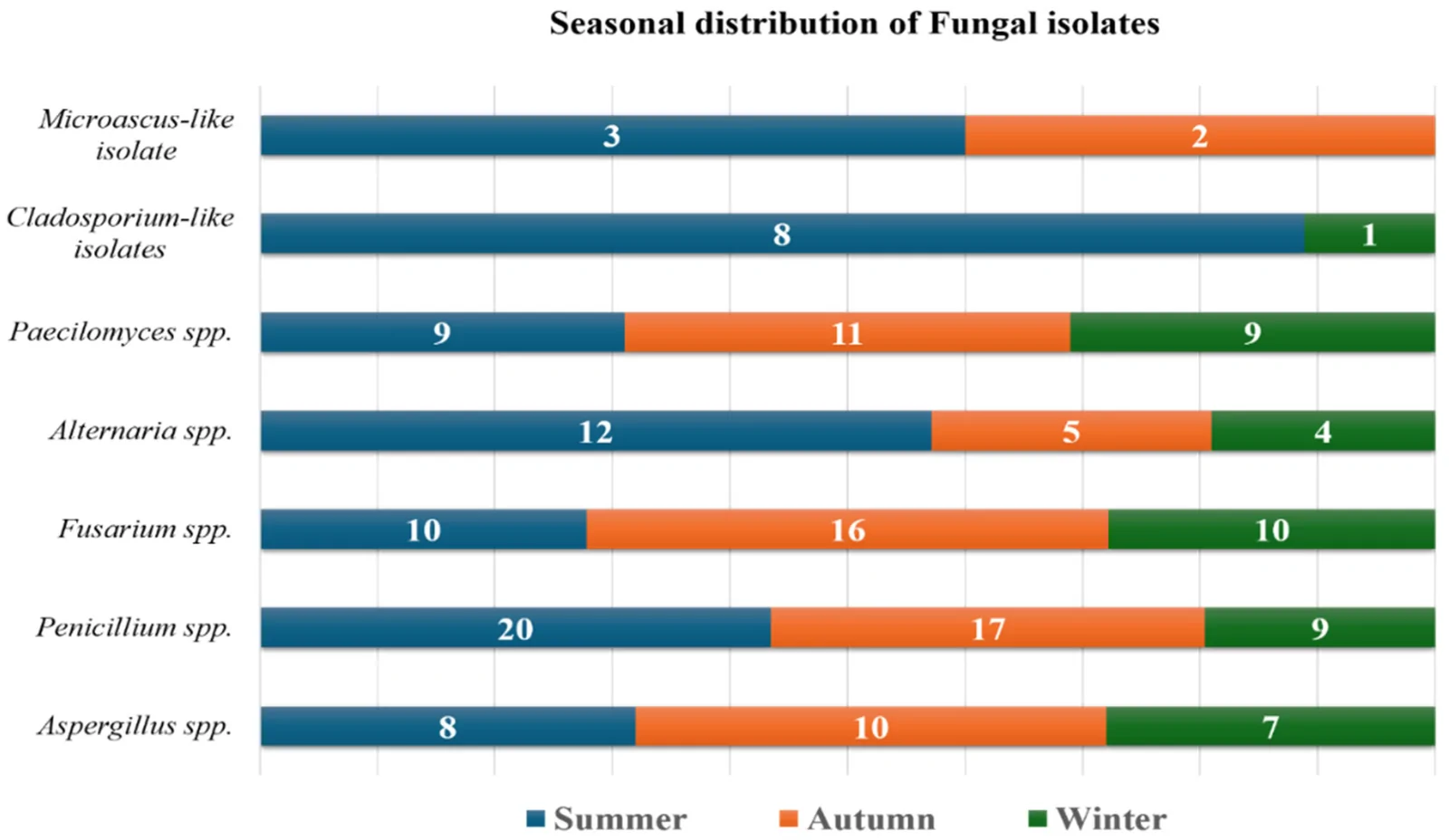
Descriptive seasonal distribution of fungal isolates recovered from Caciocavallo cheese. Numbers within the bars indicate isolate counts recovered in summer, autumn, and winter; the data are descriptive and based on pooled isolates within each season.

**Figure 2:**
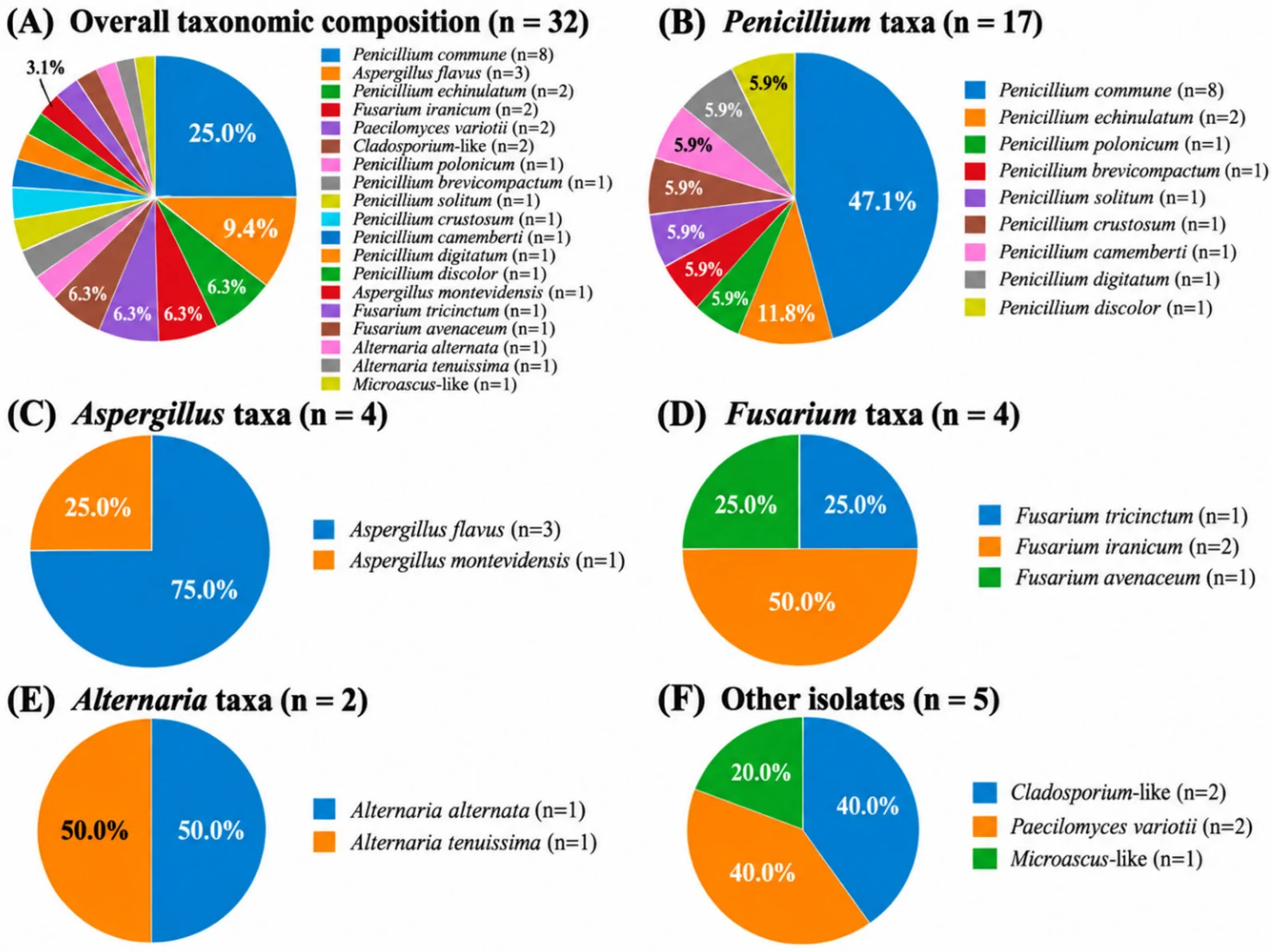
Taxonomic composition of the 32 representative fungal isolates selected for ITS and/or β-tubulin sequencing. (**A**) Overall taxonomic distribution; (**B**) *Penicillium* taxa; (**C**) *Aspergillus* taxa; (**D**) *Fusarium* taxa; (**E**) *Alternaria* taxa; and (**F**) other isolates. Percentages in panel (**A**) refer to the complete set of 32 sequenced representatives, whereas percentages in panels (**B**–**F**) are calculated within the corresponding taxonomic group. ITS only assignments in taxonomically difficult genera are considered provisional closest-match designations.

**Figure 3:**
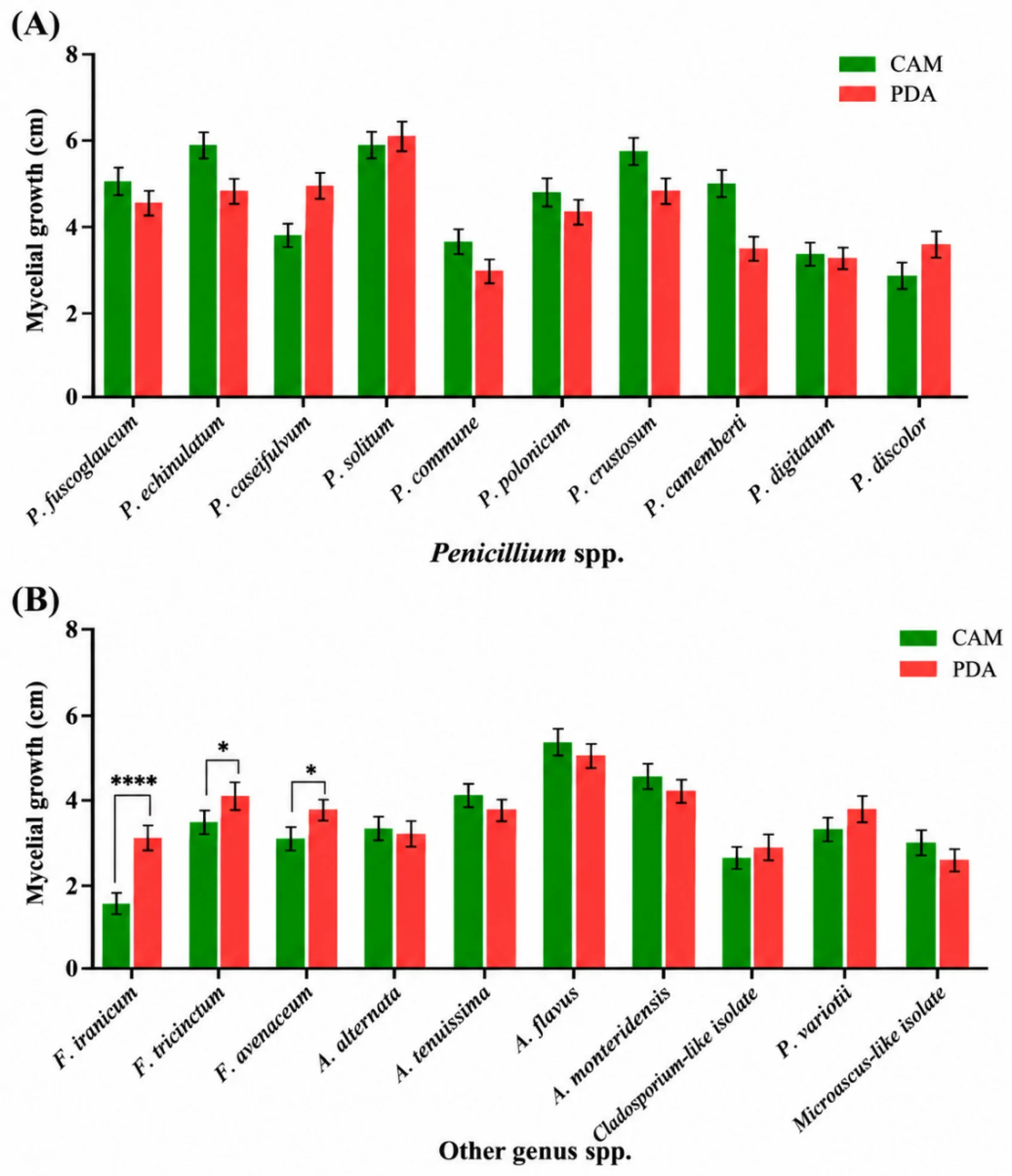
Mycelial growth (cm) of identified fungal isolates on cheese agar medium (CAM) and potato dextrose agar (PDA): (**A**) *Penicillium* isolates and (**B**) other genera. Bars show mean ± SE from three independent experiments. Asterisks indicate significant differences between CAM and PDA for the same isolate after sidak correction (* adjusted *p* < 0.05; **** adjusted *p* < 0.0001).

**Figure 4:**
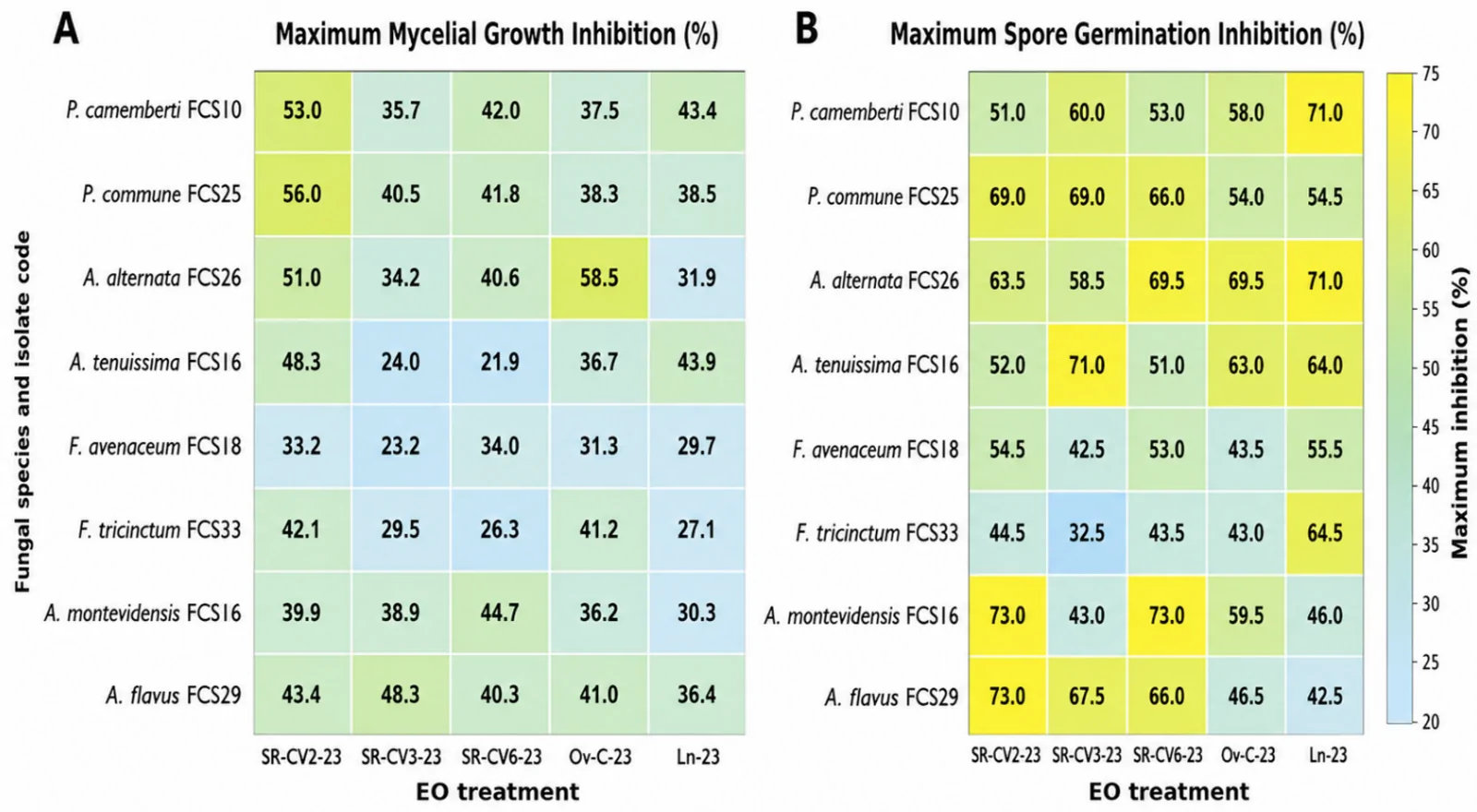
Heat maps showed the maximum inhibition (%) produced by each EO treatment at 0.35% (*v*/*v*) against representative cheese-associated fungi. (**A**) Mycelial-growth inhibition and (**B**) spore-germination inhibition. Darker yellow indicates greater inhibition, whereas blue indicates lower inhibition.

**Figure 5:**
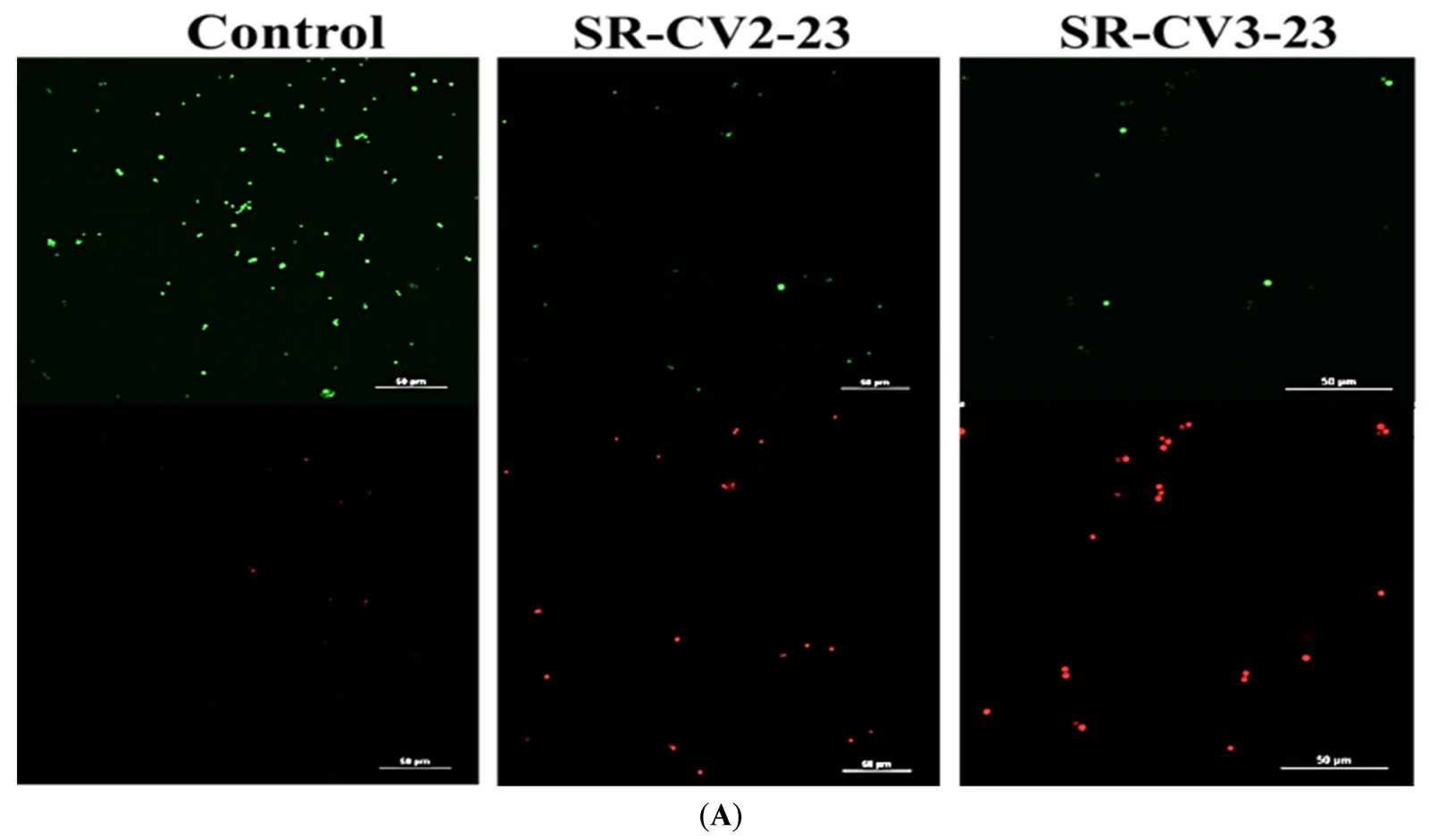

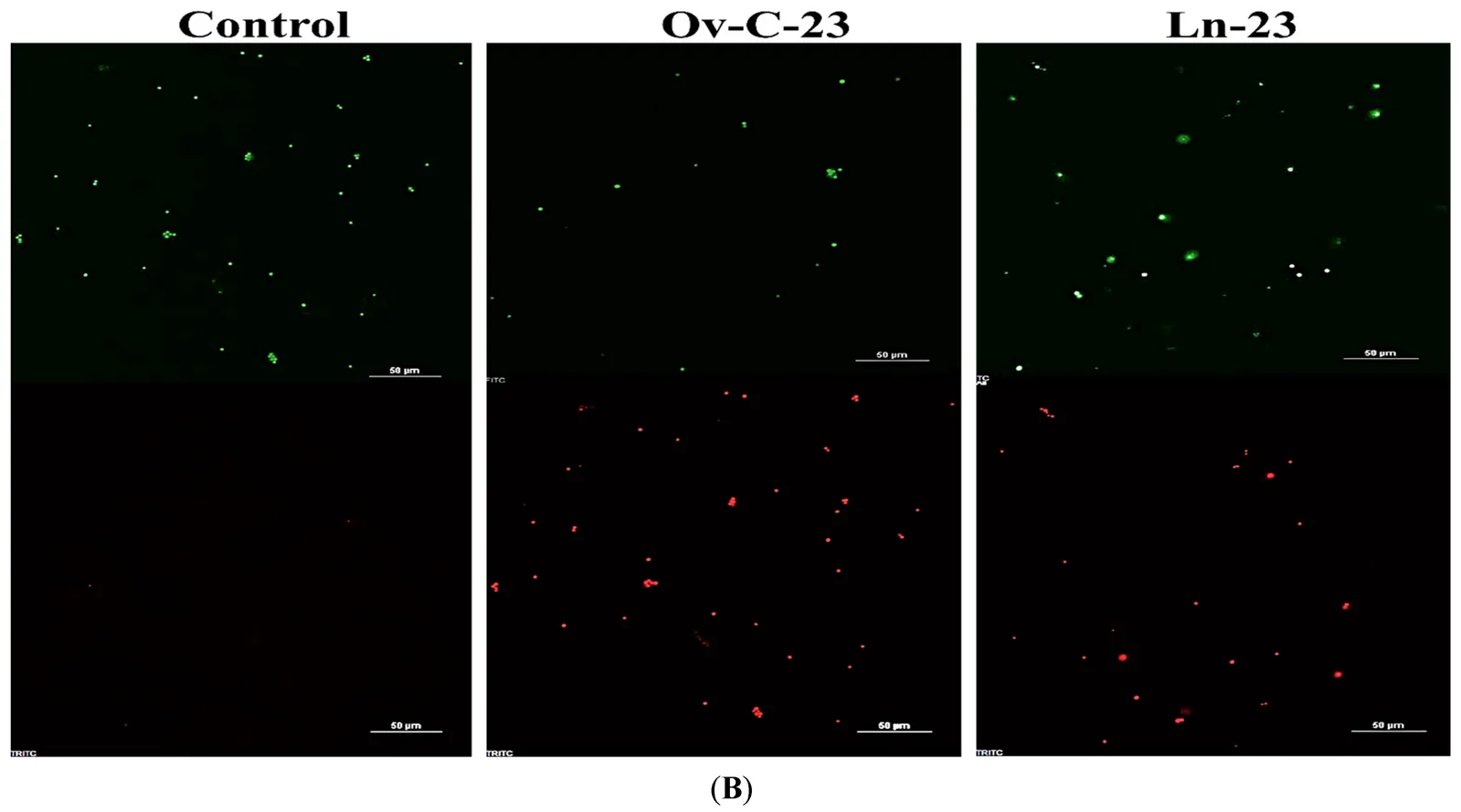
Confocal CFDA/PI fluorescence imaging of *Penicillium commune* spores after EO treatment. (**A**) Confocal images of untreated spores and spores treated with SR-CV2-23 or SR-CV3-23 at 0.35% (*v*/*v*). (**B**) Confocal images of untreated spores and spores treated with Ov-C-23 or Ln-23 at 0.35% (*v*/*v*). CFDA-associated green fluorescence indicates metabolic activity (live cell), whereas PI-associated red fluorescence indicates loss of membrane integrity (dead cell). Images are presented as qualitative comparative observations. Scale bars = 50 μm.

**Figure 6:**
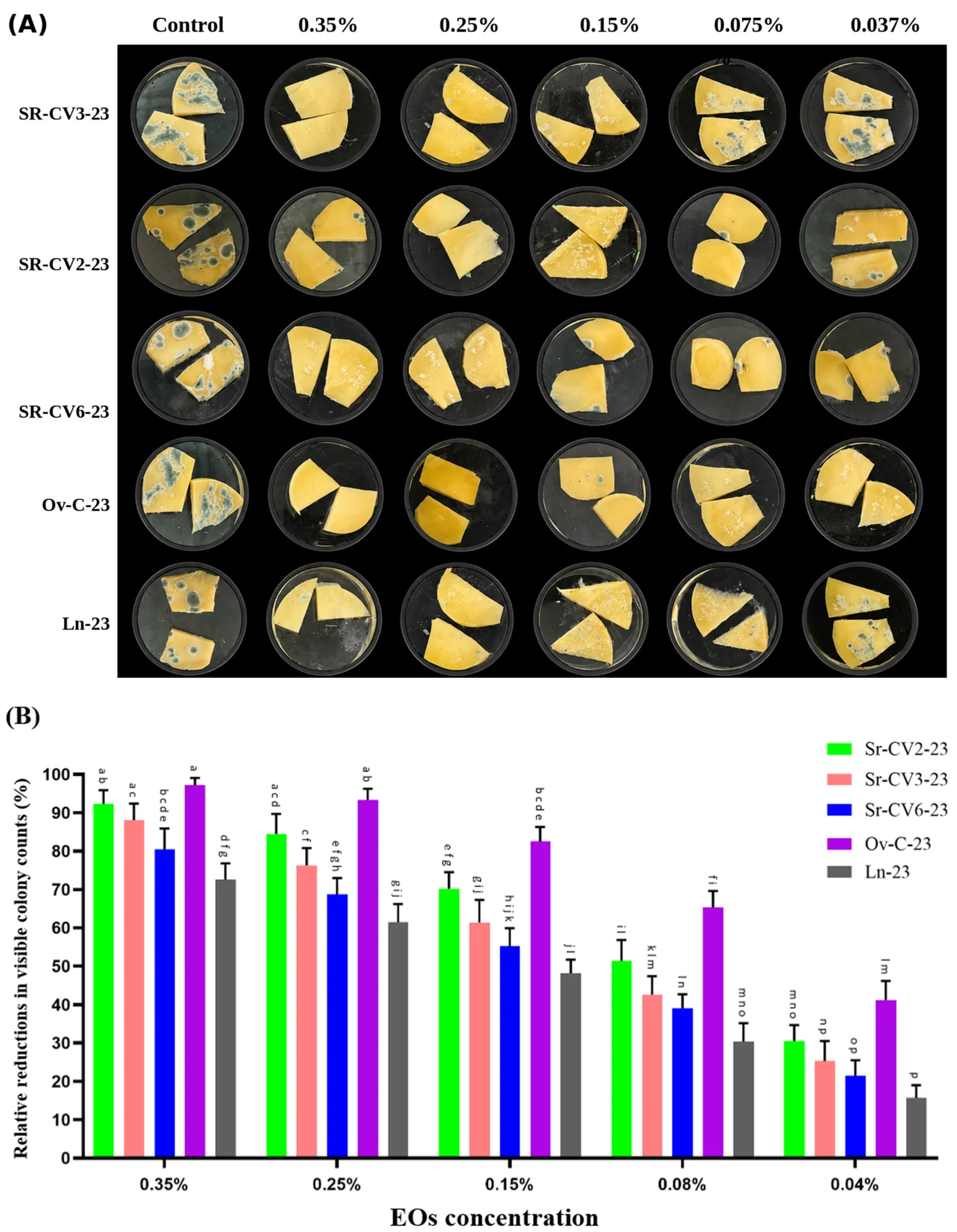
Antifungal activity of EO treatments on artificially inoculated Caciocavallo cheese pieces. (**A**) Representative images of *P. commune* colony development after 14 days at 14 ± 2 °C. (**B**) Reduction (%) in visible colonies relative to the emulsifier treated inoculated control. Bars show mean ± SE from three independent experiments. Different lowercase letters indicate significant differences among treatments (Tukey test, *p* < 0.05).

**Figure 7:**
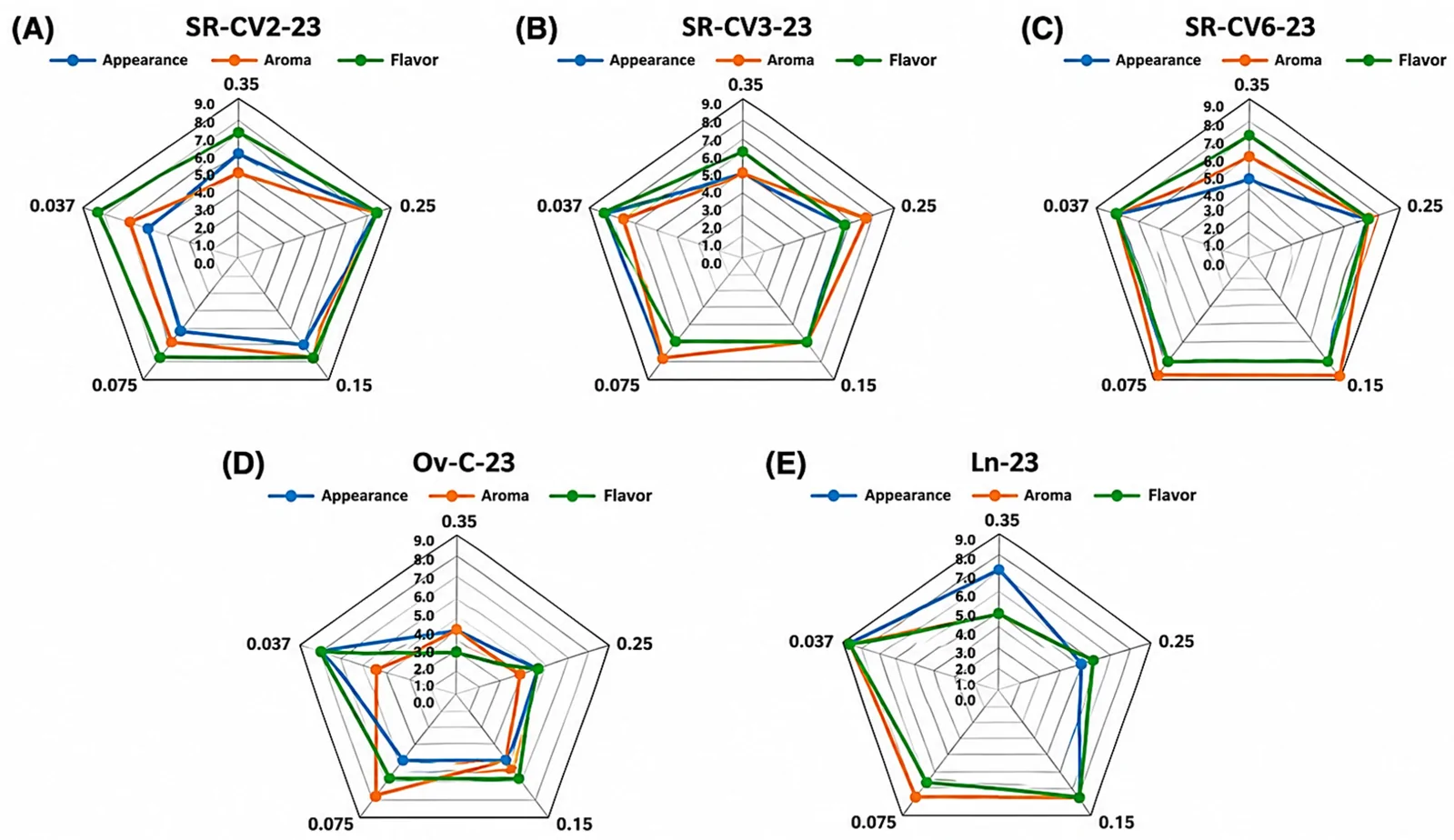
Radar plots from the preliminary sensory evaluation of Caciocavallo cheese slices treated with 0.35%, 0.25%, 0.15%, 0.075%, or 0.037% (*v*/*v*) EO. (**A**) SR-CV2-23, (**B**) SR-CV3-23, (**C**) SR-CV6-23, (**D**) Ov-C-23, and (**E**) Ln-23. Appearance, aroma, flavor, and texture were scored by 16 untrained adult assessors using a 9-point hedonic scale.

**Table 1:** Seasonal distribution of recovered fungal isolates from Caciocavallo cheese, expressed as number of isolates and descriptive frequency (%).

Fungal Genus	Summer No. of Isolates	Summer Frequency (%)	Autumn No. of Isolates	Autumn Frequency (%)	Winter No. of Isolates	Winter Frequency (%)
*Aspergillus* spp.	8	11.43	10	16.39	7	17.50
*Penicillium* spp.	20	28.57	17	27.87	9	22.50
*Fusarium* spp.	10	14.29	16	26.23	10	25.00
*Alternaria* spp.	12	17.14	5	8.20	4	10.00
*Paecilomyces* spp.	9	12.86	11	18.03	9	22.50
*Cladosporium* spp.	8	11.43	0	0.00	1	2.50
*Microascus* spp.	3	4.29	2	3.28	0	0.00
Total	70	100.00	61	100.00	40	100.00

**Table 2:** Molecular characterization of representative fungal isolates from Caciocavallo cheese, including primer pair, amplicon size, sequence identity, and the selected GenBank reference match.

Sample Code	No. of Isolates in Morphotype Group	Provisional Taxonomic Assignment	Primer Pair	bp	Identity (%)	Selected GenBank Reference Match Accession
EBT236R30	3	*Penicillium commune*	βt2a-βt2b	349	98.25	MK682843.1
ABT26R26	2	*Penicillium commune*	βt2a-βt2b	373	99.50	KT313381.1
PT437R14	4	*Penicillium commune*	ITS1-ITS4	512	99.45	KY646438.1
OT431R13	3	*Penicillium commune*	ITS1-ITS4	510	99.63	MZ047535.1
LT427R10	2	*Penicillium commune*	ITS1-ITS4	516	100.00	MK660341.1
NBT130R37	4	*Penicillium commune*	βt1a-βt1b	339	98.86	AY654875.1
LBT10R35	3	*Penicillium commune*	βt1a-βt1b	393	98.78	AY654875.1
MBT126R36	2	*Penicillium echinulatum*	βt1a-βt1b	388	98.25	JN112032.1
VBT228R20	3	*Penicillium echinulatum*	βt2a-βt2b	332	98.62	AY674342.1
YBT234R22	2	*Penicillium polonicum*	βt2a-βt2b	397	99.03	OU641499.1
ZBT229R21	3	*Penicillium brevicompactum*	βt2a-βt2b	419	99.55	PQ594437.1
IBT18R34	3	*Penicillium commune*	βt1a-βt1b	328	98.81	AY654875.1
GBT15R32	2	*Penicillium solitum*	βt1a-βt1b	398	99.76	FJ012873.1
CBT231R28	2	*Penicillium crustosum*	βt2a-βt2b	370	99.49	EU128548.1
BBT27R27	4	*Penicillium camemberti*	βt2a-βt2b	360	98.24	KY469095.1
JBT29R24	2	*Penicillium digitatum*	βt2a-βt2b	330	99.11	KF303075.1
MT42R11	2	*Penicillium discolor*	ITS1-ITS4	460	99.57	OW984227.1
RT419R16	8	*Aspergillus flavus*	ITS1-ITS4	520	99.29	DQ467975.1
FT422R6	6	*Aspergillus flavus*	ITS1-ITS4	505	99.61	KR611589.1
DT432R4	6	*Aspergillus flavus*	ITS1-ITS4	468	99.79	PX715396.1
UBT23R19	5	*Aspergillus montevidensis*	βt2a-βt2b	342	99.41	MZ826403.1
ET442R5	9	*Fusarium tricinctum*	ITS1-ITS4	413	100.00	OP596509.1
BT423R2	7	*Fusarium iranicum*	ITS1-ITS4	388	98.57	OR758512.1
GT420R7	11	*Fusarium avenaceum*	ITS1-ITS4	484	99.44	ON819345.1
HT421R8	9	*Fusarium iranicum*	ITS1-ITS4	418	99.32	OR758512.1
IT425R9	11	*Alternaria alternata*	ITS1-ITS4	497	100.00	KT223345.1
CT424R3	10	*Alternaria tenuissima*	ITS1-ITS4	414	99.12	ON208445.1
QT416R15	15	*Paecilomyces variotii*	ITS1-ITS4	493	99.02	PX760174.1
AT418R1	14	*Paecilomyces variotii*	ITS1-ITS4	409	97.95	PX760174.1
TT22R18	5	*Cladosporium*-like isolate (morphological); molecular assignment unresolved	ITS1-ITS2	677	97.31	AY293067.2
ST212R17	4	*Cladosporium*-like isolate	ITS1-ITS2	789	97.70	AY251077.2
FBT115R31	5	*Microascus*-like isolate	βt1a-βt1b	389	91.25	DQ368606.1

Note: No. of isolates in morphotype group is the total group size and includes the sequenced representative; these values sum to 171. Species names based only on ITS in taxonomically difficult genera are treated as provisional closest-match assignments rather than definitive multi-locus identifications. The final column reports reference accessions used for BLAST comparison, not accession numbers assigned to sequences generated in this study.

**Table 3:** Essential oil yield (% *w*/*w*, dry-weight basis) of the investigated plant materials.

Species	Sample Code	EO Yield (% *w*/*w* Dry Weight)
*Origanum vulgare* L. subsp. *viridulum* (Martrin-Donos) Nyman × *O. vulgare* L. subsp. *hirtum* Ietswaar	Ov-C-23	2.65
*Laurus nobilis* L.	Ln-23	1.08
*Salvia rosmarinus* Spenn.	SR-CV2-23	3.33
	SR-CV3-23	2.71
	SR-CV6-23	2.25

**Table 4:** Major constituents (%) of essential oils identified by GC-MS.

Essential Oil	Major Compound 1	%	Major Compound 2	%	Major Compound 3	%
*Salvia rosmarinus* (SR-CV2-23)	Camphor	37.83	α-Pinene	20.82	1,8-Cineole	13.29
*Salvia rosmarinus* (SR-CV3-23)	Camphor	47.84	α-Pinene	17.81	1,8-Cineole	9.26
*Salvia rosmarinus* (SR-CV6-23)	Camphor	23.81	α-Pinene	17.42	1,8-Cineole	11.35
oregano cv. Carva (Ov-C-23)	Carvacrol	83.95	p-Cymene	3.28	γ-Terpinene	3.25
*Laurus nobilis* (Ln-23)	1,8-Cineole	16.87	Linalool	14.71	Bornyl acetate	10.73

**Table 5:** Maximum inhibition of mycelial growth and spore germination by 0.35% (*v*/*v*) EOs from the three *Salvia rosmarinus* cultivars.

Fungal Species	Cultivar with Highest Observed Mycelial Inhibition	Mycelial Inhibition (%)	Cultivar with Highest Observed Spore-Germination Inhibition	Spore Inhibition (%)
*P. camemberti*	SR-CV2-23	53.0 ± 2.0	SR-CV3-23	60.0 ± 6.0
*P. commune*	SR-CV2-23	56.0 ± 2.0	SR-CV2-23 SR-CV3-23	69.0 ± 2.0 69.0 ± 3.0
*A. alternata*	SR-CV2-23	51.0 ± 2.0	SR-CV6-23	69.5 ± 3.5
*A. tenuissima*	SR-CV2-23	48.3 ± 3.7	SR-CV3-23	71.0 ± 5.0
*F. avenaceum*	SR-CV6-23	34.0 ± 5.9	SR-CV2-23	54.5 ± 1.5
*F. tricinctum*	SR-CV2-23	42.1 ± 2.0	SR-CV2-23	44.5 ± 2.5
*A. montevidensis*	SR-CV6-23	44.7 ± 2.7	SR-CV2-23 SR-CV6-23	73.0 ± 3.0
*A. flavus*	SR-CV3-23	48.3 ± 3.7	SR-CV2-23	73.0 ± 6.0

**Table 6:** Maximum inhibition of mycelial growth and spore germination by 0.35% (*v*/*v*) essential oils of *Oregano cv. Carva* (Ov-C-23) and *Laurus nobilis* L. (Ln-23).

Fungal Species	EO with Highest Observed Mycelial Inhibition	Mycelial Inhibition (%)	Best Essential Oil for Spore Inhibition	Spore Inhibition (%)
*P. camemberti*	Ln-23	43.4 ± 2.6	Ln-23	71.0 ± 6.0
*P. commune*	Ln-23	38.5 ± 1.6	Ln-23	54.5 ± 3.5
*A. alternata*	Ov-C-23	58.5 ± 4.4	Ln-23	71.0 ± 3.0
*A. tenuissima*	Ln-23	43.9 ± 2.1	Ln-23	64.0 ± 2.0
*F. avenaceum*	Ov-C-23	31.3 ± 1.8	Ln-23	55.5 ± 2.5
*F. tricinctum*	Ov-C-23	41.2 ± 1.8	Ln-23	64.5 ± 3.5
*A. montevidensis*	Ov-C-23	36.2 ± 0.4	Ov-C-23	59.5 ± 6.5
*A. flavus*	Ov-C-23	41.0 ± 1.0	Ov-C-23	46.5 ± 1.5

Note: Values are expressed as mean ± SE. Table 5 and Table 6 report the maximum inhibition observed for each fungal species and response variable. Complete concentration-dependent data are provided in Supplementary Tables S4 and S5.

**Table 7:** Primers used for PCR amplification and corresponding cycling conditions.

Target Gene/Region	Primer Set	PCR Cycling Conditions
Internal Transcribed Spacer (ITS) region	ITS1-ITS4/ITS1-ITS2	Initial denaturation: 95 °C for 5 min; 35 cycles of 95 °C for 30 s, 55 °C for 1 min, 72 °C for 1 min; final extension: 72 °C for 10 min
β-tubulin gene	βt1a-βt1b/βt2a-βt2b	Initial denaturation: 94 °C for 5 min; 35 cycles of 94 °C for 1 min, 52 °C for 1 min, 72 °C for 1 min; final extension: 72 °C for 7 min

## Data Availability

The data supporting the findings of this study are provided in the article and its Supplementary Materials.

## Supplementary Materials

The following supporting information can be downloaded at: https://www.mdpi.com/article/10.3390/molecules31193439/s1. Table S1: Chemical composition of *Salvia rosmarinus* L. essential oils (SR-CV2-23, SR-CV3-23, and SR-CV6-23) determined by GC–MS analysis; Table S2: Chemical composition of Ov-C-23 determined by GC–MS analysis; Table S3: Chemical composition of *Laurus nobilis* L. essential oil (Ln-23) determined by GC–MS analysis; Table S4: Mycelial growth and spore germination inhibition (%) of eight fungal species treated with *Salvia rosmarinus* chemotypes at different concentrations; Table S5: Mycelial growth and spore germination inhibition (%) of eight fungal species treated with *Origanum vulgare* and *Laurus nobilis* essential oils at different concentrations.
