# Supplementary material for: Antifungal Efficacy and Chemical Profiles of Salvia, Oregano, and Laurus Essential Oils Against Mold Species Associated with Caciocavallo Cheese Spoilage
Source: Molecules. 2026 Sep 27;31(19):3439. doi: 10.3390/molecules31193439 (PMC13666195; doi:10.3390/molecules31193439)
Supplement: Supplementary file 1 [file molecules-31-03439-s001.zip › Supplementary_Tables_S1-S3_FINAL.pdf]

Supplementary Table S1. Chemical composition of *Salvia rosmarinus* L. essential oils (SR-CV2-23, SR-CV3-23, and SR-CV6-23) determined by GC–MS analysis.

No.	Compound	RT (min) mean	RI exp.	RI lit. (Adams)	SR-CV2-23 (%)	SR-CV3-23 (%)	SR-CV6-23 (%)	ID
1	Tricyclene	9.10	926	921	0.22	0.20	0.16	MS, RI
2	α-Pinene	9.59	938	939	20.82	17.81	17.42	MS, RI
3	Camphene	10.11	955	954	6.41	4.67	4.27	MS, RI
4	β-Pinene	11.17	977	979	1.32	0.55	0.56	MS, RI
5	α-Phellandrene	12.27	1001	1002	0.55	0.99	1.37	MS, RI
6	δ-2-Carene	12.76	999	1001	0.99	0.91	1.17	MS, RI
7	o-Cymene	13.07	1021	1022	–	1.06	–	MS, RI
8	p-Cymene	13.08	1025	1024	1.33	–	1.73	MS, RI
9	Limonene	13.23	1027	1029	–	3.74	–	MS, RI
10	1.8-Cineole	13.33	1032	1033	13.29	9.26	11.35	MS, RI
11	γ-Terpinene	14.40	1059	1060	1.81	0.85	1.19	MS, RI
12	α-Terpinene	15.52	1014	1015	2.52	–	1.13	MS, RI
13	Linalool	16.11	1099	1098	0.83	–	–	MS, RI
14	α-Campholenal	16.97	1123	1122	0.32	–	–	MS, RI
15	Camphor	17.50	1142	1143	37.83	47.84	23.81	MS, RI, Co-I
16	Pinocamphone	18.19	1164	1165	0.1	0.25	0.24	MS
17	Pinocarvone	18.26	1163	1164	–	–	0.18	MS, RI
18	Borneol	18.40	1167	1166	0.77	4.31	5.61	MS, RI
19	Terpinen-4-ol	18.83	1176	1177	0.67	0.85	–	MS, RI
20	α-Terpineol	19.32	1188	1189	–	–	1.77	MS, RI
21	Myrtenol	19.54	1191	1193	0.08	–	0.42	MS, RI
22	Verbenone	19.98	1206	1204	1	–	8.17	MS, RI
23	Carveol	20.35	1220	1217	0.3	–	–	MS, RI
24	Carvone	21.20	1240	1242	0.10	–	–	MS, RI
25	Bornyl acetate	22.68	1289	1287	0.72	–	4.53	MS, RI
26	p-Thymol	23.10	1294	1290	0.15	–	0.13	MS, RI
27	α-Ylangene	25.52	1371	1373	–	–	0.09	MS, RI
28	Caryophyllene	27.06	1416	1418	0.10	–	1.45	MS, RI
29	α-Cedrene	27.46	1412	1414	0.38	–	–	MS, RI
30	α-Humulene	28.14	1455	1452	0.06	–	1.39	MS, RI
32	γ--Curcumene	29.05	1481	1481	0.12	–	–	MS, RI
33	β-Bisabolene	29.84	1508	1505	0.21	–	–	MS, RI
34	Sesquiphellandrene	30.29	1519	1521	0.03	–	–	MS, RI
				**Total identified compounds (%)**	**93.03**	**93.29**	**88.14**	

Abbreviations: *RT, retention time; RI exp., experimental retention index; RI lit., literature retention index; MS, mass-spectrum matching; Co-I, co-injection with an authentic standard. Dash (–) indicates not detected.*

Supplementary Table S2. Chemical composition of Ov-C-23 determined by GC–MS analysis.

No.	Compound	RT (min)	RI exp.	RI lit. (Adams)	Area (%)	ID
1	α-Thujene	12.93	929	924	0.85	MS, RI
2	α-Pinene	13.24	938	939	0.35	MS, RI
3	Camphene	14.00	955	954	0.06	MS, RI
4	Sabinene	15.42	977	975	0.05	MS, RI
5	Oct-3-ol	16.08	986	984	0.17	MS, RI
6	β-Myrcene	16.41	989	991	1.01	MS, RI
7	α-Phellandrene	17.03	1001	1002	0.16	MS, RI
8	δ-3-Carene	17.31	1012	1011	0.05	MS, RI
9	α-Terpinene	17.67	1015	1017	0.68	MS, RI
10	p-Cymene	18.14	1025	1024	3.28	MS, RI
11	β-Phellandrene	18.32	1029	1030	0.23	MS, RI
12	β-Ocimene	18.90	1043	1044	0.04	MS, RI
13	γ-Terpinene	19.91	1059	1060	3.25	MS, RI
14	Sabinene hydrate	20.46	1069	1068	0.61	MS, RI
15	Linalool oxide	21.55	1073	1074	0.05	MS, RI
16	Linalool	22.17	1099	1098	0.23	MS, RI
17	Borneol	25.49	1165	1166	0.16	MS, RI
18	Terpinen-4-ol	26.00	1178	1177	0.34	MS, RI
19	α-Terpineol	26.71	1190	1189	0.11	MS, RI
20	Carvacrol methyl ether	29.12	1244	1245	0.06	MS, RI
21	Thymol	31.79	1288	1290	0.34	MS, RI, Co-I
22	Carvacrol	32.26	1301	1298	83.95	MS, RI, Co-I
23	trans-Caryophyllene	36.78	1421	1418	2.61	MS, RI
24	α-Humulene	38.21	1450	1452	0.22	MS, RI
25	β-Bisabolene	40.41	1508	1505	0.36	MS, RI
26	Caryophyllene oxide	43.44	1587	1583	0.15	MS, RI
				**Total identified compounds (%)**	**96.72**	

Abbreviations: *RT, retention time; RI exp., experimental retention index; RI lit., literature retention index; MS, mass-spectrum matching; Co-I, co-injection with an authentic standard. Dash (–) indicates not detected.*

Supplementary Table S3. Chemical composition of *Laurus nobilis* L. essential oil (Ln-23) determined by GC–MS analysis.

No.	Compound	RT (min)	RI exp.	RI lit. (Adams)	Area (%)	ID
1	α-Thujene	8.61	929	924	0,81	MS, RI
2	α-Pinene	8.85	938	939	6,41	MS, RI
3	Camphene	9.33	955	954	1,42	MS, RI
4	Sabinene	10.39	977	975	7,23	MS, RI
5	β-Pinene	10.44	981	979	2,63	MS, RI
6	β-Myrcene	11.07	989	991	0,78	MS, RI
7	α-Phellandrene	11.47	1001	1002	0,29	MS, RI
8	3-Carene	11.69	1012	1011	0,75	MS, RI
9	α-Terpinene	11.96	1015	1017	0,67	MS, RI
10	p-Cymene	12.27	1025	1024	0,42	MS, RI
11	1,8-Cineole	12.60	1032	1033	16,87	MS, RI, Co-I
12	γ-Terpinene	13.58	1059	1060	1,08	MS, RI
13	α-Terpinolene	14.67	1089	1088	0,36	MS, RI
14	Linalool	15.39	1099	1098	14,71	MS, RI, Co-I
15	Camphor	16.70	1142	1143	0,27	MS, RI
16	Borneol	17.53	1165	1166	0,86	MS, RI
17	Terpinen-4-ol	17.99	1176	1177	2,47	MS, RI
18	Estragole	18.74	1196	1195	0,21	MS, RI
19	Verbenone	19.07	1205	1204	0,18	MS, RI
20	(Z)-Geraniol	19.82	1249	1250	0,36	MS, RI
21	Linalyl acetate	20.80	1255	1257	0,63	MS, RI

22	4-Thujen-2-yl acetate	21.44	1268	1265	0,13	MS, RI
23	Bornyl acetate	23.72	1275	1272	10,73	MS, RI
24	Eugenol	24.32	1354	1356	7,07	MS, RI
25	β-Elemene	25.28	1392	1389	0,44	MS, RI
26	Methyleugenol	25.78	1407	1403	3,00	MS, RI
27	trans-Caryophyllene	26.11	1416	1418	0,71	MS, RI
28	Aromadendrene	26.72	1439	1441	0,26	MS, RI
29	α-Guaiene	27.39	1441	1440	0,32	MS, RI
30	trans-α-Bergamotene	28.17	1434	1432	0,89	MS, RI
31	β-Selinene	28.50	1488	1485	0,36	MS, RI
32	δ-Cadinene	29.31	1520	1522	0,21	MS, RI
33	Elemicin	30.34	1558	1555	0,18	MS, RI
34	Spathulenol	30.88	1575	1578	0,25	MS, RI
35	β-Selinelol	32.93	1653	1651	0,29	MS, RI
**Total identified compounds (%)**					**84,25**	

Abbreviations: *RT, retention time; RI exp., experimental retention index; RI lit., literature retention index; MS, mass-spectrum matching; Co-I, co-injection with an authentic standard. Dash (–) indicates not detected.*
