# Supplementary material for: Antifungal Efficacy and Chemical Profiles of Salvia, Oregano, and Laurus Essential Oils Against Mold Species Associated with Caciocavallo Cheese Spoilage
Source: Molecules. 2026 Sep 27;31(19):3439. doi: 10.3390/molecules31193439 (PMC13666195; doi:10.3390/molecules31193439)
Supplement: Supplementary file 1 [file molecules-31-03439-s001.zip › Supplementary_Tables_S4-S5_FINAL.pdf]

Supplementary Table S4. Mycelial growth and spore germination inhibition (%) of eight fungal species treated with *Salvia rosmarinus* chemotypes at different concentrations.

Chemotype	Conc. (% v/v)	Parameter	*P. camemberti*	*P. commune*	*A. alternata*	*A. tenuissima*	*F. avenaceum*	*F. tricinctum*	*A. montevidensis*	*A. flavus*
**SR-CV2-23**	**0.35**	**Mycelial**	53.0 ± 2.0	56.0 ± 2.0	51.0 ± 2.0	48.3 ± 3.7	33.2 ± 2.0	42.1 ± 2.0	39.9 ± 0.8	43.4 ± 1.6
	0.35	Spore	51.0 ± 4.0	69.0 ± 2.0	63.5 ± 3.5	52.0 ± 2.0	54.5 ± 1.5	44.5 ± 2.5	73.0 ± 3.0	73.0 ± 6.0
	0.25	Mycelial	50.0 ± 2.0	51.5 ± 0.5	34.6 ± 3.5	32.1 ± 3.0	29.7 ± 2.3	41.3 ± 0.7	38.3 ± 2.3	35.6 ± 1.5
	0.25	Spore	48.5 ± 3.5	67.0 ± 3.0	63.0 ± 3.0	48.0 ± 1.0	49.5 ± 2.5	37.5 ± 0.5	68.5 ± 2.5	70.0 ± 2.0
	0.15	Mycelial	44.5 ± 2.5	45.5 ± 2.5	28.8 ± 3.2	29.4 ± 1.1	20.4 ± 1.6	39.1 ± 0.9	35.1 ± 2.9	30.6 ± 1.4
	0.15	Spore	45.5 ± 1.5	62.5 ± 2.5	58.5 ± 1.5	45.0 ± 1.0	45.5 ± 2.5	30.0 ± 2.0	61.5 ± 5.5	62.0 ± 2.0
	0.075	Mycelial	42.0 ± 2.0	41.0 ± 2.0	27.2 ± 2.8	24.5 ± 1.5	16.7 ± 1.3	37.3 ± 1.7	25.5 ± 1.5	21.7 ± 2.3
	0.075	Spore	45.0 ± 2.0	54.5 ± 1.5	52.5 ± 2.5	38.5 ± 3.5	43.0 ± 1.0	28.5 ± 2.5	56.0 ± 2.0	58.0 ± 2.0
	0.037	Mycelial	40.9 ± 1.1	38.5 ± 2.5	26.3 ± 0.7	23.3 ± 1.6	13.9 ± 1.4	30.1 ± 2.0	24.8 ± 1.8	20.2 ± 0.9
	0.037	Spore	26.5 ± 1.5	37.0 ± 5.0	45.5 ± 1.5	32.5 ± 5.5	38.5 ± 1.5	26.0 ± 1.0	50.5 ± 0.5	50.0 ± 2.0
**SR-CV3-23**	**0.35**	**Mycelial**	35.7 ± 4.3	40.5 ± 3.4	34.2 ± 2.8	24.0 ± 2.0	23.2 ± 1.7	29.1 ± 3.9	38.9 ± 3.5	48.3 ± 3.7
	0.35	Spore	60.0 ± 6.0	69.0 ± 3.0	57.5 ± 7.5	71.0 ± 5.0	42.5 ± 3.5	32.5 ± 4.5	43.0 ± 3.0	67.5 ± 4.5
	0.25	Mycelial	31.2 ± 1.8	34.5 ± 2.6	31.3 ± 2.0	22.7 ± 1.9	20.2 ± 1.0	29.5 ± 2.4	35.1 ± 2.9	43.3 ± 1.7
	0.25	Spore	48.0 ± 2.0	62.5 ± 2.5	58.5 ± 1.5	65.5 ± 4.5	40.0 ± 2.0	28.5 ± 4.5	34.5 ± 2.5	59.0 ± 3.0
	0.15	Mycelial	26.8 ± 1.2	29.6 ± 1.4	27.8 ± 2.2	19.9 ± 1.5	17.2 ± 1.1	27.4 ± 2.6	30.3 ± 3.2	39.2 ± 0.8
	0.15	Spore	55.5 ± 1.5	58.0 ± 4.0	43.0 ± 3.0	65.0 ± 3.0	33.5 ± 3.5	24.5 ± 2.5	29.5 ± 2.5	54.0 ± 2.0
	0.075	Mycelial	23.3 ± 3.7	25.7 ± 2.3	26.6 ± 1.4	14.2 ± 2.9	15.5 ± 1.7	21.4 ± 3.0	28.2 ± 2.9	37.9 ± 2.1
	0.075	Spore	42.0 ± 2.0	54.0 ± 4.0	51.5 ± 2.5	44.0 ± 2.0	29.0 ± 4.0	20.0 ± 2.0	25.5 ± 3.5	45.0 ± 2.0
	0.037	Mycelial	20.0 ± 2.0	21.3 ± 2.7	20.0 ± 1.8	7.7 ± 1.3	13.6 ± 2.0	15.0 ± 2.0	22.3 ± 2.7	33.6 ± 2.4
	0.037	Spore	40.0 ± 2.0	45.5 ± 1.5	51.0 ± 6.0	38.5 ± 0.5	19.0 ± 3.0	17.0 ± 2.0	19.5 ± 1.5	31.5 ± 1.5
**SR-CV6-23**	**0.35**	**Mycelial**	42.0 ± 2.0	41.8 ± 2.2	40.6 ± 5.5	21.9 ± 1.5	34.0 ± 5.9	26.3 ± 1.6	44.7 ± 2.7	40.3 ± 1.7
	0.35	Spore	53.0 ± 2.0	66.0 ± 1.0	69.5 ± 3.5	51.0 ± 3.0	53.0 ± 3.0	43.5 ± 3.5	73.0 ± 3.0	66.0 ± 3.0
	0.25	Mycelial	36.2 ± 0.9	34.9 ± 5.1	35.4 ± 3.5	17.0 ± 0.6	33.5 ± 3.4	21.4 ± 2.5	36.9 ± 3.1	33.9 ± 1.6
	0.25	Spore	49.0 ± 3.0	57.5 ± 4.5	58.0 ± 2.0	47.0 ± 2.0	49.0 ± 3.0	35.5 ± 2.5	63.5 ± 3.5	57.5 ± 4.5
	0.15	Mycelial	30.2 ± 1.0	27.0 ± 3.1	27.2 ± 2.9	12.7 ± 1.4	30.0 ± 5.3	17.5 ± 4.4	28.0 ± 2.0	28.5 ± 1.5
	0.15	Spore	45.5 ± 1.5	53.0 ± 3.0	57.0 ± 3.0	44.0 ± 2.0	32.0 ± 2.0	29.5 ± 2.5	61.0 ± 1.0	49.5 ± 2.5
	0.075	Mycelial	25.2 ± 2.8	21.8 ± 3.2	21.5 ± 1.2	10.1 ± 3.7	16.6 ± 2.2	12.9 ± 3.2	22.8 ± 3.2	22.7 ± 2.3
	0.075	Spore	31.0 ± 1.0	49.5 ± 4.5	52.5 ± 2.5	36.5 ± 1.5	30.5 ± 0.5	31.5 ± 6.5	55.0 ± 3.0	44.5 ± 1.5
	0.037	Mycelial	20.2 ± 1.8	15.6 ± 2.5	17.9 ± 0.7	9.6 ± 2.6	11.6 ± 1.5	11.2 ± 2.5	17.0 ± 2.0	16.0 ± 1.1
	0.037	Spore	24.0 ± 1.0	35.0 ± 3.0	45.5 ± 1.5	26.0 ± 1.0	24.0 ± 2.0	25.5 ± 1.5	49.5 ± 1.5	41.0 ± 1.0

Note. Values are expressed as mean ± SE.

Supplementary Table S5. Mycelial growth and spore germination inhibition (%) of eight fungal species treated with *Origanum vulgare and Laurus nobilis essential oils at different concentrations.*

**Chemotype**	**Conc. (% v/v)**	**Parameter**	***P. camemberti***	***P. commune***	***A. alternata***	***A. tenuissima***	***F. avenaceum***	***F. tricinctum***	***A. montevidensis***	***A. flavus***
**Ov-C-23**	**0.35**	**Mycelial**	37.5 ± 1.6	38.3 ± 1.7	58.5 ± 4.4	36.7 ± 1.3	31.3 ± 1.8	41.2 ± 1.8	36.2 ± 0.4	41.0 ± 1.0
	0.35	Spore	58.0 ± 1.0	54.0 ± 2.0	69.5 ± 2.5	63.0 ± 3.0	43.5 ± 3.5	43.0 ± 1.0	59.5 ± 6.5	46.5 ± 1.5
	0.25	Mycelial	35.0 ± 2.0	34.8 ± 2.6	53.1 ± 2.9	27.8 ± 2.2	29.4 ± 1.7	35.8 ± 2.4	32.6 ± 1.3	38.5 ± 1.5
	0.25	Spore	51.0 ± 1.0	49.0 ± 1.0	59.5 ± 2.5	56.5 ± 3.5	30.0 ± 3.0	36.5 ± 1.5	49.5 ± 2.5	43.5 ± 1.5
	0.15	Mycelial	28.7 ± 1.3	31.7 ± 3.2	49.4 ± 2.1	18.2 ± 1.8	26.8 ± 2.2	33.8 ± 2.2	28.9 ± 7.4	36.0 ± 2.0
	0.15	Spore	46.0 ± 2.0	44.0 ± 1.0	52.5 ± 2.5	51.5 ± 3.5	23.5 ± 3.5	36.5 ± 3.5	45.0 ± 2.0	40.5 ± 3.5
	0.075	Mycelial	24.3 ± 1.8	26.5 ± 2.0	45.0 ± 3.2	15.7 ± 2.4	24.1 ± 2.4	27.4 ± 2.4	30.3 ± 5.4	31.8 ± 2.8
	0.075	Spore	36.0 ± 6.0	40.5 ± 3.5	51.0 ± 5.0	49.0 ± 2.0	20.0 ± 2.0	28.5 ± 1.5	41.0 ± 1.0	36.5 ± 3.5
	0.037	Mycelial	20.3 ± 2.0	24.6 ± 2.4	39.8 ± 2.2	17.4 ± 1.3	16.0 ± 1.0	15.6 ± 2.0	33.5 ± 4.9	29.5 ± 2.8
	0.037	Spore	32.0 ± 5.0	30.5 ± 2.5	43.5 ± 0.5	33.0 ± 2.0	12.5 ± 5.5	25.5 ± 3.5	34.5 ± 2.5	30.0 ± 2.0
**Ln-23**	**0.35**	**Mycelial**	43.4 ± 2.6	38.5 ± 1.6	31.9 ± 2.1	43.9 ± 2.1	29.7 ± 2.3	27.1 ± 1.9	30.3 ± 2.7	36.4 ± 2.6
	0.35	Spore	71.0 ± 6.0	54.5 ± 3.5	71.0 ± 3.0	64.0 ± 2.0	55.5 ± 2.5	64.5 ± 3.5	46.0 ± 2.0	42.5 ± 2.5
	0.25	Mycelial	38.5 ± 0.6	34.4 ± 0.6	22.8 ± 2.4	31.6 ± 1.4	25.0 ± 1.1	23.5 ± 1.5	27.5 ± 2.5	28.4 ± 2.7
	0.25	Spore	54.5 ± 4.5	48.0 ± 2.0	66.5 ± 1.5	56.5 ± 2.5	53.0 ± 3.0	63.0 ± 1.0	42.5 ± 1.5	40.0 ± 3.0
	0.15	Mycelial	37.3 ± 2.7	32.6 ± 2.6	16.3 ± 1.7	29.5 ± 1.3	21.6 ± 1.3	20.6 ± 1.4	26.7 ± 1.7	25.8 ± 2.2
	0.15	Spore	62.5 ± 2.5	43.0 ± 1.0	54.5 ± 1.5	51.5 ± 1.5	50.0 ± 2.0	48.5 ± 2.5	38.5 ± 1.5	37.5 ± 2.5
	0.075	Mycelial	26.7 ± 3.3	23.2 ± 0.8	14.6 ± 1.5	24.6 ± 2.5	19.7 ± 1.1	17.5 ± 1.5	25.6 ± 1.7	26.5 ± 0.6
	0.075	Spore	54.0 ± 2.0	32.5 ± 2.5	52.0 ± 2.0	48.5 ± 0.5	44.5 ± 2.5	40.0 ± 2.0	32.0 ± 2.0	31.5 ± 1.5
	0.037	Mycelial	22.7 ± 3.4	19.7 ± 1.4	13.8 ± 0.5	17.8 ± 2.1	18.6 ± 1.5	13.5 ± 1.5	23.3 ± 1.2	24.6 ± 1.4
	0.037	Spore	43.0 ± 3.0	25.0 ± 2.0	46.5 ± 1.5	43.0 ± 3.0	42.0 ± 2.0	39.0 ± 3.0	32.0 ± 2.0	30.5 ± 1.5

Note. Values are expressed as mean ± SE.
